# Discovery of a Kojibiose Hydrolase by Analysis of Specificity-Determining Correlated Positions in Glycoside Hydrolase Family 65

**DOI:** 10.3390/molecules26206321

**Published:** 2021-10-19

**Authors:** Emma De Beul, Alana Jongbloet, Jorick Franceus, Tom Desmet

**Affiliations:** Centre for Synthetic Biology (CSB), Department of Biotechnology, Ghent University, Coupure Links 653, 9000 Ghent, Belgium; emma.debeul@ugent.be (E.D.B.); alana.jongbloet@gmail.com (A.J.); jorick.franceus@ugent.be (J.F.)

**Keywords:** Glycoside Hydrolase Family 65, glycoside phosphorylases, glycoside hydrolases, correlated positions, CMA, specificity determinants, kojibiase, kojibiose glucohydrolase

## Abstract

The Glycoside Hydrolase Family 65 (GH65) is an enzyme family of inverting α-glucoside phosphorylases and hydrolases that currently contains 10 characterized enzyme specificities. However, its sequence diversity has never been studied in detail. Here, an in-silico analysis of correlated mutations was performed, revealing specificity-determining positions that facilitate annotation of the family’s phylogenetic tree. By searching these positions for amino acid motifs that do not match those found in previously characterized enzymes from GH65, several clades that may harbor new functions could be identified. Three enzymes from across these regions were expressed in *E. coli* and their substrate profile was mapped. One of those enzymes, originating from the bacterium *Mucilaginibacter mallensis*, was found to hydrolyze kojibiose and α-1,2-oligoglucans with high specificity. We propose kojibiose glucohydrolase as the systematic name and kojibiose hydrolase or kojibiase as the short name for this new enzyme. This work illustrates a convenient strategy for mapping the natural diversity of enzyme families and smartly mining the ever-growing number of available sequences in the quest for novel specificities.

## 1. Introduction

**Figure 1:**
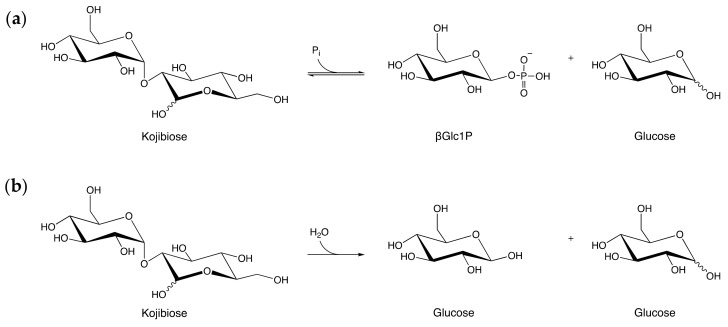
The two types of reactions catalyzed by enzymes in the Glycoside Hydrolase Family 65 (GH65) illustrated by their respective reactions on kojibiose: (**a**) Reaction catalyzed by kojibiose phosphorylase; (**b**) Reaction catalyzed by kojibiose hydrolase. P_i_: inorganic phosphate, βGlc1P: β-d-glucose 1-phosphate.

In the carbohydrate-active enzymes database (CAZy) [1], 10 enzyme specificities (Table 1) are grouped to form the Glycoside Hydrolase Family 65 (GH65), which is a member of clan GH-L. All members of this enzyme family break α-glucosidic bonds through a single displacement mechanism that inverts the anomeric configuration (Figure 1), but they differ in the substrates they prefer [2]. Based on the nucleophile that is used in the breakdown reaction, two groups can be distinguished: glucoside hydrolases use water to hydrolyze the glucosidic bond, whereas glucoside phosphorylases employ inorganic phosphate, releasing glucose or β-glucose 1-phosphate (βGlc1P) as a reaction product, respectively [3]. GH65 enzymes are typically active on α-glucobioses (trehalose, kojibiose, nigerose or maltose) or derivatives thereof (trehalose 6-phosphate, α-1,2- or α-1,3-oligoglucans), but a few other natural substrates have also been identified (3-*O*-α-glucosyl-l-rhamnose, 2-*O*-α-glucosylglycerol or the α-glucosyl-1,2-β-galactosyl decoration found on hydroxylysine residues of collagen) [2,4,5,6].

In the past, efforts have been made to elucidate how substrate specificity is controlled in GH65 enzymes. Yamamoto et al. constructed several chimeric enzymes of the kojibiose phosphorylase (KP) and trehalose phosphorylase (TP) of *Thermoanaerobacter brockii*. Surprisingly, a certain chimera of 785 amino acids contained only one segment of 125 residues originating from the KP (Met384–Thr512) but still exhibited KP activity. This region, ranging from α3 to α6 of the (α,α)_6_-barrel catalytic domain, was therefore suspected to contain crucial residues for substrate recognition [34]. Within this region, Nakai et al. identified loop 3, a loop that connects α3 and α4 and forms the rim of the active site, as a potential specificity determinant. Loop 3 is typically conserved within one specificity but highly divergent, both in length and amino acid sequence, between different specificities. Through inspection of the crystal structure and mutational analysis, they were able to identify a three-residue motif within this loop (His413, Asn414 and Glu415 in *Lactobacillus acidophilus* maltose phosphorylase) that is crucial for maltose binding. Replacing this HNE-motif with the corresponding residues in the KP (TPK) or TP (SAY) of *Thermoanaerobacter brockii* severely impaired the phosphorolytic activity on maltose, while introducing a low activity on kojibiose or trehalose, respectively [35]. Later, Okada et al. determined the crystal structure of the KP of *Caldicellulosiruptor saccharolyticus*, which resulted in the discovery of another substrate recognition motif. Three residues (Trp391, Glu392 and Thr417) within loop 3 that bind the glucose moiety of kojibiose in the +1 subsite, are highly conserved within all KPs, but show different motifs in other specificities. However, a specificity switch could not be induced by changing the WET-motif into the patterns observed in maltose-, trehalose- or nigerose-active enzymes [4].

Both motifs identified by Nakai et al. and Okada et al. were based on manual inspection of multiple sequence alignments (MSAs) and analysis of crystal structures. Although they were able to identify crucial residues for maltose resp. kojibiose binding, an in-depth search for specificity determinants of the entire GH65 family has never been performed, as it would be practically impossible to investigate the entire sequence diversity with these ad-hoc methods. However, in the context of sequence annotation, enzyme discovery and specificity engineering, it would be extremely beneficial to identify certain positions in the family’s MSA that can be used as an indicator for specificity. Those so-called specificity-determining positions are conserved among all enzymes that share the same specificity but mutate simultaneously across specificity boundaries. If such positions exist, they should be easily picked up by correlated mutations analysis (CMA), a type of statistical analysis that identifies positions in an MSA that underwent interdependent mutations through evolution. As substrate binding is a complex interplay of different residues and interactions, one can expect that a certain function-switching mutation requires one or more compensatory mutations for the protein to remain highly active. These co-evolving positions will thus form a correlation network that can be detected in an MSA through CMA [36].

This technique was already successfully used in the past to uncover specificity fingerprints. Glucokinases could be distinguished from other hexokinases based on six correlated positions that surround the active site [37]. In a similar manner, a network of nine correlated positions was detected in the isocitrate lyase and phosphoenolpyruvate mutase superfamily. One of those nine positions always housed a serine residue in oxaloacetate hydrolases (OAH) and could thus be used as a very specific marker for the OAH subfamily [37]. Bacterial lytic polysaccharide monooxygenases in the Auxiliary Activity Family 10 with activity on cellulose either oxidize exclusively the C1-carbon, or both the C1- and C4-carbon of their substrate. A co-evolving network of 13 positions was shown to be a reliable indicator for this variation in oxidative regioselectivity [38,39]. Recently, we described the so-called heptagonal box model for the NDP-sugar active short-chain dehydrogenase/reductase superfamily based on conservation and correlation patterns. The different subfamilies and specificities can be distinguished based on which amino acids occupy the seven “walls” or fingerprint regions of the model [40].

In this work, we used CMA to identify specificity-determining positions for the GH65 family. Visualization of this specificity fingerprint on the family’s phylogenetic tree did not only facilitate functional annotation of the sequences in this family, but also uncovered a new enzyme specificity. We describe the discovery of a kojibiose glucohydrolase from the bacterium *Mucilaginibacter mallensis*. Our work demonstrates the potential of CMA for mapping and exploring the natural diversity of an enzyme family and can be beneficial for further explorative endeavors.

## 2. Results

### 2.1. Correlated Mutations Analysis

**Figure 2:**
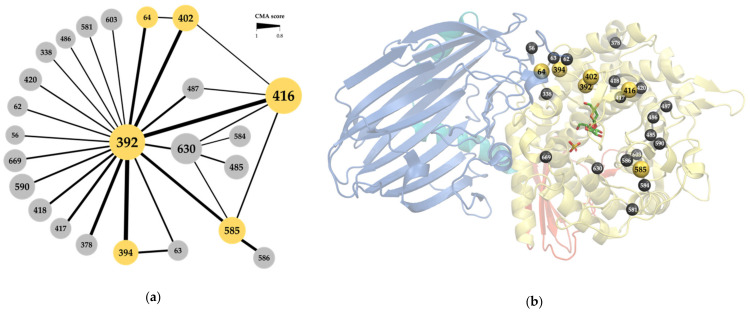
(**a**) Correlated position network of the GH65 family alignment, determined with Comulator [37]. Nodes represent the alignment positions, numbering according to the kojibiose phosphorylase of *Caldicellulosiruptor saccharolyticus* (*Cs*KP). Node size indicates the number of edges. Edge thickness indicates the strength of pair-wise correlation. The six selected positions are highlighted in yellow; (**b**) Structure of *Cs*KP (PDB ID: 3WIQ) with the location of the correlated positions (yellow and gray spheres). The four structural domains are indicated in different colors: N-terminal domain (blue), linker region (cyan), catalytic domain (yellow) and C-terminal domain (red). Kojibiose (green sticks) and sulphate (yellow sticks) are also shown.

All sequences in family GH65 were extracted from the CAZy database to build a family alignment. In what follows, the sequence numbering of the kojibiose phosphorylase of *Caldicellulosiruptor saccharolyticus* (*Cs*KP) will be used to refer to MSA positions, unless stated otherwise. CMA was performed with Comulator [37], which revealed 24 positions in the MSA with a correlation score of 0.8 or higher (Figure 2a). Position 392 clearly holds a central position in the correlation network, with 20 co-evolutionary interactions (CMA ≥ 0.8) detected. All correlated positions are part of the (α,α)_6_-barrel catalytic domain, except for positions 56, 62, 63 and 64, which are located in a loop that emanates from the N-terminal β-sandwich and is known to be involved in the active site architecture of the *Levilactobacillus brevis* maltose phosphorylase (Figure 2b) [4]. Some of these residues are found within the active site, while others are further away, with the distance between their α-carbon and that of the catalytic acid ranging from 5 to 27 Å. For reasons of clarity and simplicity, this selection of correlated positions was further narrowed down. Since both the correlation strength, measured as the CMA score, and the number of correlated partners are important indicators for overall correlation [36], only the six positions with at least one CMA score higher than 0.9 and at least two correlation partners (CMA ≥ 0.8) will be focused on in the rest of the analysis, namely positions 64, 392, 394, 402, 416 and 585.

### 2.2. Correlated Positions as Specificity Determinants

**Figure 3:**
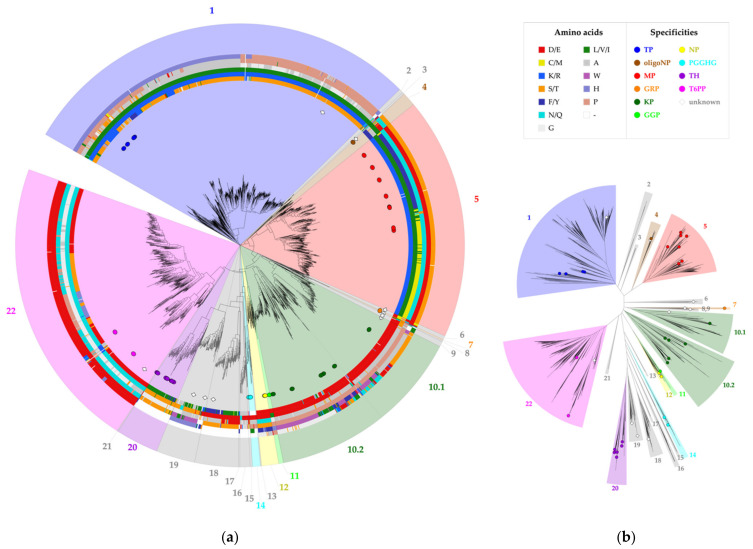
Phylogenetic tree of family GH65. All annotated representatives and all new enzymes discussed in this study are indicated with circles and diamonds, respectively. The tree is divided into 22 subgroups, which are colored according to their putative specificity. The legend specifies the colors used for each amino acid and each enzyme specificity. (**a**) Circular tree representation with a visualization of the amino acids present at six correlated positions, shown as colored rings around the phylogenetic tree. From inside to outside, these rings represent positions 64, 392, 394, 402, 416 and 585 (*Cs*KP numbering); (**b**) Unrooted tree representation.

**Figure 4:**
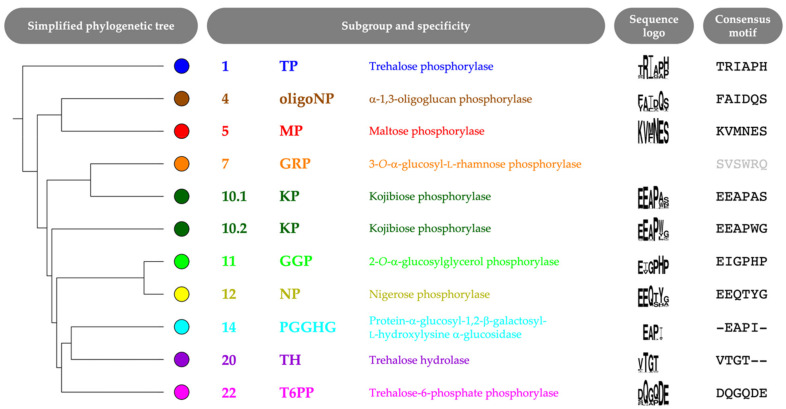
Simplified phylogenetic tree with sequence logo and consensus motif of six correlated positions (left to right, positions 64, 392, 394, 402, 416 and 585; *Cs*KP numbering) for each subgroup that contains characterized GH65 enzymes. A sequence logo for subgroup 7 is not shown, as this branch contains only one sequence (with motif SVSWRQ).

The selected correlated positions were visualized on the family’s phylogenetic tree by means of colored rings (Figure 3, Figure S1). The combination of coevolutionary and phylogenetic information was used to divide the entire GH65 family into 22 subgroups. Subgroups that contain enzymes that have previously been characterized experimentally (Table 1) were annotated with the reported substrate specificity. Taking a closer look at the sequence logos of the correlated positions for these subgroups, it is clear that a comparison of the conserved motifs can easily distinguish all specificities (Figure 4, Figure S2). For example, maltose phosphorylases (MPs) have a highly conserved KV[MF]NES motif, whereas the EEAPxx motif is characteristic of KPs.

To verify the predictive power of these motifs to act as specificity indicators, four enzymes were selected from different subgroups that already contain annotated representatives. Sequences originating from thermophilic organisms that reside in functionally uncharacterized branches of the subgroups were preferred. The sequences of choice originated from the organisms *Thermobispora bispora* (*Tb*GP, GenBank ID: ADG89586.1, in subgroup 1), *Caldicellulosiruptor hydrothermalis* (*Ch*GP, GenBank ID: ADQ05832.1, in subgroup 4), *Halothermothrix orenii* (*Ho*GP, GenBank ID: ACL68803.1, in subgroup 10.1) and *Caldithrix abyssi* (*Ca*GP, GenBank ID: APF18594.1, in subgroup 22). Based on their location in the tree and their specificity motifs (TRIGPP, FAITQA, EEAPWS and DQGQDE), they were predicted to be a trehalose phosphorylase (TP), an α-1,3-oligoglucan phosphorylase (oligoNP), a kojibiose phosphorylase (KP) and a trehalose-6-phosphate phosphorylase (T6PP), respectively. All four enzymes were expressed in *E. coli* and purified by means of their His_6_-tag with a yield of 20.2, 41.6, 40.5 and 5.1 mg protein from lysates of a 250 mL-culture, respectively. Their acceptor profile was evaluated by screening them on 46 potential substrates (Table S1). The enzymes were found to show the predicted activity (Figure S3). In a reaction mixture of glucose and βGlc1P, *Tb*GP produced trehalose. *Ch*GP elongated nigerose, and other disaccharides, to form α-1,3-oligoglucans. *Ho*GP showed typical KP behavior, as it was able to elongate both mono- and disaccharides with α-1,2-bound glucose units. In a reaction mixture that contained both kojibiose and βGlc1P, phosphorolytic and synthetic reactions co-occur, both breaking kojibiose down to glucose and βGlc1P and using it as acceptor to produce α-1,2-glucans up to a degree of polymerization (DP) of 6. Finally, our prediction for *Ca*GP was also confirmed, as trehalose 6-phosphate was produced from βGlc1P and glucose 6-phosphate.

### 2.3. Specificity-Determining Correlated Positions as Tool for Enzyme Discovery

Subgroups 2, 3, 6, 8, 9, 13, 15–19 and 21 could not be annotated as they do not contain any characterized enzymes. Moreover, their sequence motifs at the specificity-determining correlated positions diverge significantly from those of already described enzymes (Figure S2). These subgroups might contain enzymes with new properties or even new enzyme specificities, and are therefore interesting candidates for further exploration. Six enzymes were selected from such subgroups. Based on the residues that occupy positions known to be involved in phosphate binding [4,41,42], three of them were predicted to be phosphorylases, whereas the other three were predicted to be hydrolases (Figure S4). The three putative glycoside phosphorylases originate from the bacteria *Mageeibacillus indolicus* (*Mi*GP, GenBank ID: ADC90669.1, in subgroup 6), *Kiritimatiella glycovorans* (*Kg*GP, GenBank ID: AKJ64725.1, in subgroup 8) and *Paenibacillus riograndensis* (*Pr*GP, GenBank ID: CQR58226.1, in subgroup 9). The three putative hydrolases originate from the bacteria *Mucilaginibacter mallensis* (*Mm*GH, GenBank ID: SDT07729.1, in subgroup 18), *Phyllobacterium zundukense* (*Pz*GH, GenBank ID: ATU91641.1, in subgroup 19) and *Streptomyces* sp. (*Stre*GH, GenBank ID: AEN12156.1, in subgroup 19). Soluble expression of *Kg*GP, *Pz*GH and *Stre*GH in *E. coli* was not successful, and these enzymes were thus not further investigated. However, *Mi*GP, *Pr*GP and *Mm*GH did express well and were purified by means of their C-terminal His_6_-tag, with a yield of 6.2, 17.7 and 26.5 mg protein from lysates of a 250 mL-culture, respectively. The acceptor profile of the putative phosphorylases *Mi*GP and *Pr*GP was mapped by screening them in the synthesis direction of the reversible reaction on 46 potential acceptor substrates (Table S1). Both enzymes showed activity towards a diverse set of substrates. Apart from minor activity on glucose and galactose, *Mi*GP prefers α-glucosidic disaccharides, whereas *Pr*GP is mainly active on monosaccharides, even on some l-sugars. The results of this acceptor screening did however not give a clear hint for the natural activity of *Mi*GP and *Pr*GP and their true specificity remains a mystery for now.

The substrate profile of the predicted hydrolase *Mm*GH was evaluated by screening its activity on nine α-glucosides as potential substrates. The enzyme showed very high activity on kojibiose and weak activity on nigerose, but was not capable of breaking down trehalose, maltose, isomaltose, sucrose, isomaltulose, turanose or melezitose (Table 2). *Mm*GH was also able to hydrolyze α-1,2-oligoglucans with a higher DP (Figure S5). Based on these results it was hypothesized that kojibiose might be the natural substrate of *Mm*GH (Figure 1b).

### 2.4. Optimal pH and Temperature and Kinetic Properties of MmGH

**Figure 5:**
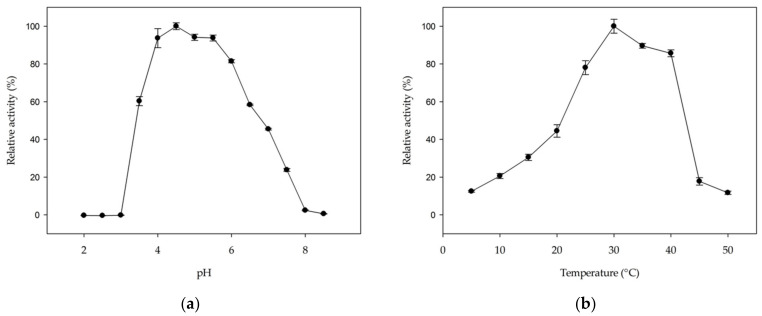
The effect of (**a**) pH and (**b**) temperature on the hydrolytic activity of *Mm*GH on kojibiose. Error bars represent standard error from three independent experiments.

The hydrolytic activity of *Mm*GH on kojibiose is optimal in a pH range from 4 to 5.5 (Figure 5a). The optimal pH range is comparable to that of other hydrolases in family GH65 [27,28,32,33,43,44,45], but differs from GH65 phosphorylases, which typically prefer neutral pH values [5,14,18,19,22,25,26,41]. The optimal temperature was found to be 30 °C (Figure 5b), which is at the higher end of the growth range of *Mucilaginibacter mallensis* (optimal growth at 25 °C) [46].

The kinetic parameters of *Mm*GH were determined at the optimal pH (4.5) and temperature (30 °C). The enzyme exhibited Michaelis–Menten kinetics under these conditions, and *K*_M_ and *k*_cat_ values of 0.77 ± 0.01 mM and 9.9 ± 0.3 s^−1^ were deduced. The catalytic efficiency (*k*_cat_/*K*_M_) equals 13 mM^−1^ s^−1^. The affinity of *Mm*GH for kojibiose is higher than that of other GH65 hydrolases for their preferred substrate (*K*_M_ values between 2.6 and 5.7 mM) [31,33,43,45], which further substantiates that kojibiose is indeed the true substrate of this novel hydrolase.

## 3. Discussion

In this study, we used CMA to uncover distinct sequence patterns that were applied as specificity fingerprints. Our approach allowed us to analyze the entire GH65 family in a systematic, rather than ad-hoc, manner. This is especially important in light of the continuously growing number of sequences available in databases. For instance, the GH65 family contained 1520 sequences in the CAZy database in 2015 [47], whereas this number has increased to 8189 in 2021 (29 August 2021). In earlier work, we already discovered two new enzyme specificities based on rational comparison of sequence motifs, but information about structure–function relationships was required as an input [48,49]. Here, we report how CMA is a relatively easy method to detect specificity-determining positions in large datasets without any prior knowledge required. The described strategy should be readily applicable to other protein families.

The possible applications of CMA are manifold. Firstly, we showed how analysis of correlated mutations allowed protein annotation and phylogenetic tree analysis. Conservation patterns can be used as an indicator for specificity, which can help to predict the activity of unknown sequences and to identify homologues of a certain protein of interest. Visualizing the correlated positions on the phylogenetic tree resulted in an informative and easy-to-read figure, which facilitated annotation of clades in the tree. Furthermore, the identified specificity fingerprint was also demonstrated to be relevant for enzyme discovery. Guided by the conservation patterns, we discovered a dedicated hydrolase for the breakdown of kojibiose. Future efforts for the discovery of novel enzymes in GH65 could focus on elucidating the natural activity of *Mi*GP and *Pr*GP, and the other unexplored subgroups (2, 3, 13, 15–17 and 21) are of particular interest as well. Finally, CMA might also be valuable for enzyme engineering endeavors, as it provides insight into non-obvious interactions between residues that would not be easily exposed by manual analysis of sequences and crystal structures [36]. The finding that certain positions are entangled in a co-evolving network should sound a cautionary note for mutating these residues in engineering studies. Substituting an amino acid at one position might require compensatory mutations in other positions in the network. Unwittingly disturbing this network can have a dramatic impact on the enzyme’s functionality. This could possibly explain why earlier attempts to mutate positions 392, 402 and 417 of the GH65 correlation network resulted in severely impaired catalytic activity [4,35].

The analysis of correlated positions guided the discovery of a kojibiose hydrolase, for which no EC number is currently available. A few glucosidases have been reported to show some hydrolytic activity on kojibiose, but they typically have a rather relaxed substrate specificity and kojibiose is never their preferred substrate (Table S2). To the best of our knowledge, *Mm*GH is the first glucosidase that is highly specific for kojibiose. We therefore propose kojibiose glucohydrolase as the systematic name and kojibiose hydrolase or kojibiase as the short name for this new enzyme. Kojibiose is now the second sugar, next to trehalose, for which both a dedicated hydrolase and phosphorylase exist in the GH65 family. Therefore, these would make interesting model enzymes for investigating the evolutionary relationship between glycoside hydrolases and phosphorylases [50], even though they only show 15–25% sequence identity. Besides its activity on kojibiose, *Mm*GH is also able to act on α-1,2-oligoglucans, an ability it shares with its phosphorylase counterparts [20,21,22,23]. *Mm*GH’s side-activity on nigerose (0.22% compared to kojibiose) did not come as a surprise either, as KPs have been reported to phosphorolyze nigerose with a similar relative activity (0.23–0.73%) [22,23].

## 4. Materials and Methods

### 4.1. Materials

All chemicals were of analytical grade and were obtained from Sigma-Aldrich/Merck (Darmstadt, Germany) or Carbosynth (Berkshire, United Kingdom), except for psicose (Izumori Lab, Kagawa University, Kagawa, Japan), glycerol (Chem-Lab, Zedelgem, Belgium), tagatose (Nutrilab, Giessen, The Netherlands) and trehalose (Cargill, Vilvoorde, Belgium). Kojibiose, nigerose and βGlc1P were produced in-house according to the procedures of Beerens et al. [51], Franceus et al. [52] and Van der Borght et al. [53], respectively.

### 4.2. Sequence Analysis

All protein sequences classified in family GH65 (January 2020) were extracted from the CAZy database (www.cazy.org) [1]. Redundant sequences with more than 90% sequence identity were removed using CD-HIT with standard parameters [54]. Any annotated GH65 representatives (Table 1) that were removed in this step were added back to the dataset manually. The resulting sequences were aligned with Clustal Omega using default parameters [55]. All 59 sequences lacking the catalytic acid were removed, resulting in a final dataset of 1953 sequences. Those were re-aligned in two steps. First, all characterized GH65 representatives (Table 1) were structurally aligned using MAFFT-DASH [56]. Next, this MSA was used as a skeleton alignment to which the rest of the dataset was aligned with the seed-option of MAFFT [57]. All positions with a gap content of 95% or higher were removed, resulting in a final MSA of 1490 positions.

A phylogenetic tree was generated with FastTree on the BOOSTER server (https://booster.pasteur.fr/) (accessed on 12 January 2021). Branch supports were calculated as transfer bootstrap expectations (TBE) based on 200 bootstrap replicates [58]. The tree was visualized in iTOL [59]. Correlated positions were determined using the Bio-Prodict Comulator tool (https://comulator.bio-prodict.com/) (accessed on 24 January 2021) [37] and visualized as colored rings (RasMol color scheme) around the phylogenetic tree using the built-in function in iTOL. Sequence logos were generated using WebLogo 3 (http://weblogo.threeplusone.com/) (accessed on 19 May 2021) [60]. Figure 2b and Figure S4b were made with PyMOL [61].

### 4.3. Gene Cloning and Transformation

The genes encoding the enzymes expressed in this paper were codon-optimized for *E. coli* (Table S3), synthesized and subcloned into a pET21a vector at NheI and XhoI restriction sites, introducing a C-terminal His_6_-tag, by GeneArt (Thermo Fisher Scientific, Waltham, MA, USA). The plasmid was used for transformation of *E. coli* BL21(DE3) *agp*- electrocompetent cells.

### 4.4. Enzyme Expression and Purification

An overnight culture of the appropriate strain was inoculated (2 *v/v*%) in 250 mL lysogeny broth (LB) medium (10 g/L tryptone, 5 g/L yeast extract and 5 g/L NaCl) supplemented with 100 µg/mL ampicillin and incubated at 37 °C with continuous shaking at 200 rpm until the OD_600_ reached ~0.6. Subsequently, enzyme expression from the pET21a vector was induced by adding isopropyl β-d-thiogalactopyranoside (IPTG) to a final concentration of 0.1 mM and continuing incubation at 20 °C overnight. Cells were harvested by centrifugation (30 min at 9000 rpm and 4 °C). Cell pellets were frozen at −20 °C for at least one hour. For enzyme extraction and purification, the pellet of a 250 mL culture was resuspended in 8 mL of lysis buffer (10 mM imidazole, 100 µM phenylmethanesulfonyl fluoride (PMSF), 1 mg/mL lysozyme, 500 mM NaCl, 50 mM sodium phosphate, pH 7.4) and cooled on ice for 30 min. The resulting suspension was sonicated 2 times 3 min (Branson sonifier 450, level 3, 50% duty cycle). Next, the crude cell extract was separated from the cell debris by centrifugation (30 min at 9000 rpm and 4 °C) and subsequently purified by nickel-nitrilotriacetic acid (Ni-NTA) affinity chromatography. The HisPur^TM^ Ni-NTA resin (1.5 mL, Thermo Fisher Scientific) was washed with 8 mL water and equilibrated twice with 8 mL equilibration buffer (10 mM imidazole, 500 mM NaCl, 50 mM sodium phosphate, pH 7.4) before the crude cell extract was added to allow binding to the resin. Next, the resin was washed three times with 8 mL wash buffer (30 mM imidazole, 500 mM NaCl, 50 mM sodium phosphate, pH 7.4). Protein was eluted with 8 mL elution buffer (250 mM imidazole, 500 mM NaCl, 50 mM sodium phosphate, pH 7.4). The buffer was exchanged to 50 mM 3-(*N*-morpholino)propanesulfonic acid (MOPS) (pH 7.0) using a 30 kDa cut-off Amicon centrifugal filter unit (Millipore). The protein content was determined by measuring the absorbance at 280 nm with the NanoDrop 2000 spectrophotometer (Thermo Fisher Scientific). The extinction coefficient and molecular weight of His_6_-tagged enzymes were calculated using the ProtParam tool on the ExPASy server (https://web.expasy.org/protparam/) (accessed on 24 February 2021). Molecular weight and purity of the protein were verified by sodium dodecyl sulfate polyacrylamide gel electrophoresis (SDS-PAGE; 10% gel).

### 4.5. Detection of Reaction Components

Phosphorylase activity was monitored in the synthetic direction of the reversible reaction by measuring the release of inorganic phosphate using the phosphomolybdate assay described by Gawronski and Benson (2004) [62]. Hydrolytic activity was monitored by the release of glucose, which could be quantified with an enzymatic coupled assay using glucose oxidase and peroxidase (GOD-POD) [63]. The assay solution contained 0.45 mg/mL GOD, 69.2 µg/mL POD and 0.5 mg/mL 2,2′-azino-bis(3-ethylbenzthiazoline-6-sulfonic acid) (ABTS) in 1 M acetate buffer (pH 4.5). As the pH of the assay solution was not sufficient to inactivate *Mm*GH, samples (25 µL) were first inactivated with 1 M NaOH (25 µL) before adding 200 µL of the assay solution. After 30 min incubation at 37 °C, absorbance was measured at 420 nm. Reactions were also monitored by high-performance anion exchange chromatography (HPAEC), coupled to a pulsed amperometric detection (PAD) system. A Dionex ICS-3000 (Thermo Fisher Scientific) with a CarboPac PA20 pH-stable column was used. A 5-µL heat-inactivated (10 min at 95 °C) and appropriately diluted sample was analyzed at a constant flow rate of 0.5 mL/min at 30 °C. The eluent always contained 100 mM NaOH, but the concentration of sodium acetate linearly increased from 10 mM at the start to 600 mM after 18 min. This composition was maintained for 1 min, after which the acetate concentration was gradually changed back to 10 mM during 2 min. After reaching this initial composition, the run continued for 4 min.

### 4.6. Screening of Potential Substrates

The acceptor profile of the selected glycoside phosphorylases was evaluated by screening them on 46 potential acceptors (Table S4) using the phosphomolybdate assay. Reactions were performed with 10 mM βGlc1P, 10 mM of the acceptor and 0.1 mg/mL purified enzyme in 50 mM MOPS buffer (pH 7.0) at room temperature. For all hits, the same reaction was repeated at 30 °C and 1000 rpm and analyzed with HPAEC-PAD to dismiss any false positives.

For *Mm*GH, nine compounds (trehalose, kojibiose, nigerose, maltose, isomaltose, isomaltulose, sucrose, turanose and melezitose) were evaluated as potential substrates. Reactions were performed with 10 mM of the substrate and 0.5 mg/mL purified enzyme in 100 mM sodium acetate buffer (pH 4.5) at room temperature. Samples were taken every 2 min for 16 min and were analyzed with the GOD-POD assay. For kojibiose, this reaction was repeated with 5 µg/mL purified enzyme to ensure measurement of the initial velocity. For all hits, the same reaction was repeated at 30 °C and 1000 rpm and analyzed with HPAEC-PAD to dismiss any false positives. To evaluate *Mm*GH’s activity on α-1,2-oligoglucans, a mixture of kojitriose (~90%) and kojitetraose (~9%) was produced with *Cs*KP [22]. A reaction was performed with ~50 mM kojitriose, ~5 mM kojitetraose and 0.1 mg/mL purified enzyme in 100 mM sodium acetate buffer (pH 4.5) at 30 °C and 1000 rpm. Samples were analyzed with HPAEC-PAD.

### 4.7. Optimal pH and Temperature and Kinetic Properties of MmGH

The influence of pH on enzyme activity was determined in 100 mM McIlvaine buffer (pH 2.5–4.0), 100 mM sodium acetate buffer (pH 4.5–5.0), 100 mM 2-(*N*-morpholino)ethanesulfonic acid (MES) buffer (pH 5.5–6.5), 100 mM MOPS buffer (pH 7.0–7.5) or 100 mM tris(hydroxymethyl)aminomethane-hydrochloride (Tris-HCl) buffer (pH 8.0–8.5) at 30 °C. The optimal temperature was determined in 100 mM sodium acetate buffer (pH 4.5). For each reaction, 5 µg/mL purified enzyme was incubated with 50 mM kojibiose. Samples were taken every 2 min for 16 min and analyzed with the GOD-POD assay.

The apparent kinetic parameters of *Mm*GH for kojibiose were determined at the optimal temperature (30 °C) and pH (4.5) in 100 mM sodium acetate buffer. Michaelis–Menten curves were obtained using 5 µg/mL purified enzyme and varying kojibiose concentrations (0.25–10 mM). Parameters were calculated by non-linear regression of the Michaelis–Menten equation using SigmaPlot 13. The molecular weight (73.7 kDa) was used to calculate the turnover number *k*_cat_. All tests were performed in triplicate. One unit of enzyme activity was defined as the amount of enzyme hydrolyzing one µmol of substrate per minute under the specified conditions.

## Figures and Tables

**Table 1:** Overview of all currently identified GH65 enzyme specificities and their annotated representatives. Gal: d-galactose, Glc: d-glucose, Glc6P: d-glucose 6-phosphate, Hyl: 5-hydroxy-l-lysine, l-Rham: l-rhamnose, βGlc1P: β-d-glucose 1-phosphate.

EC	Enzyme	Abbreviation	Substrate	Product	Organisms with Annotated Representatives
2.4.1.8	Maltose phosphorylase	MP	Glcα1→4Glc	βGlc1P	*Levilactobacillus brevis* [7] *Fructilactobacillus sanfranciscensis* [8] *Bacillus* sp. [9] *Paenibacillus* sp. [10] *Lactobacillus acidophilus* sp. [11] *Salisediminibacterium selenitireducens* [12] *Enterococcus faecalis* [13]
2.4.1.64	Trehalose phosphorylase	TP	Glcα1→1αGlc	βGlc1P	*Thermoanaerobacter brockii* [14] *Geobacillus stearothermophilus* [15] *Caldanaerobacter subterraneus* [16] *Salisediminibacterium selenitireducens* [17]
2.4.1.216	Trehalose-6-phosphate phosphorylase	T6PP	Glcα1→1αGlc6P	βGlc1P	*Lactococcus lactis* [18] *Ca. Kuenenia stuttgartiensis* [19]
2.4.1.230	Kojibiose phosphorylase	KP	Glcα1→2Glc Glcα1→(2Glcα1→)_n-2_2Glc	βGlc1P	*Thermoanaerobacter brockii* [20,21] *Caldicellulosiruptor saccharolyticus* [22] *Nostoc* sp. [19] *Pyrococcus* sp. [23] *Escherichia coli* [24]
2.4.1.279	Nigerose phosphorylase	NP	Glcα1→3Glc	βGlc1P	*Lachnoclostridium phytofermentans* [5,19]
2.4.1.282	3-*O*-α-glucosyl-l-rhamnose phosphorylase	GRP	Glcα1→3l-Rham	βGlc1P	*Lachnoclostridium phytofermentans* [25]
2.4.1.332	2-*O*-α-glucosylglycerol phosphorylase	GGP	Glcα1→2Glycerol	βGlc1P	*Salisediminibacterium selenitireducens* [26]
2.4.1.334	α-1,3-oligoglucan phosphorylase	oligoNP	Glcα1→(3Glcα1→)_n-2_3Glc	βGlc1P	*Lachnoclostridium phytofermentans* [5]
3.2.1.28	Trehalose hydrolase (trehalase)	TH	Glcα1→1αGlc	Glc	*Saccharomyces cerevisiae* [27] *Aspergillus nidulans* [28] *Candida albicans* [29,30] *Metarhizium anisopliae* [31] *Candida parapsilosis* [32] *Candida glabrata* [33]
3.2.1.107	Protein-α-glucosyl-1,2-β-galactosyl-l-hydroxylysine α-glucosidase	PGGHG	Glcα1→2Galβ1→5Hyl	Glc	*Gallus gallus* [6] *Homo sapiens* [6]

**Table 2:** Hydrolytic activity of *Mm*GH on nine potential substrates. Specific activity is reported as mean ± standard deviation of three independent experiments. Fru: d-fructose, Glc: d-glucose, n.d.: not detected (<0.01 U/mg).

Substrate	Structure	Specific Activity (U/mg) ^1^	Relative Activity (%)
Kojibiose	Glcα1→2Glc	7.6 ± 0.3	100
Nigerose	Glcα1→3Glc	0.016 ± 0.001	0.22
Trehalose	Glcα1→1αGlc	n.d.	-
Maltose	Glcα1→4Glc	n.d.	-
Isomaltose	Glcα1→6Glc	n.d.	-
Sucrose	Glcα1→2βFru	n.d.	-
Isomaltulose	Glcα1→6Fru	n.d.	-
Turanose	Glcα1→3Fru	n.d.	-
Melezitose	Glcα1→3Fruβ2→1Glc	n.d.	-

^1^ Reactions contained 10 mM substrate in 100 mM sodium acetate buffer (pH 4.5) at 30 °C.

## Data Availability

The data are available within this article and its Supplementary Materials.

## Supplementary Materials

The following are available online, Figure S1: Phylogenetic tree of family GH65 with a visualization of the amino acids present at 24 correlated positions, Figure S2: Simplified phylogenetic tree with sequence logo of 24 correlated positions, Figure S3: Reaction profile of selected GH65 glycoside phosphorylases in the synthetic direction of the reversible reaction, Figure S4: Glycoside phosphorylases and hydrolases in family GH65, Figure S5: Hydrolysis of α-1,2-oligoglucans by *Mm*GH, Table S1: Acceptor profile of six GH65 glycoside phosphorylases in the synthetic direction of the reversible reaction, Table S2: Comparison of the substrate profile of all α-glucosidases with hydrolytic activity on kojibiose reported in the BRENDA database and *Mm*GH, Table S3: Nucleotide sequences encoding the enzymes expressed in this work, Table S4: Overview of all compounds tested as possible acceptor for the selected GH65 glycoside phosphorylases [64,65,66,67,68,69,70,71,72,73,74,75,76,77,78,79,80,81,82,83,84,85,86,87,88].

## Sample Availability

The plasmid for expression of *Mm*GH has been deposited at BCCM/GeneCorner Plasmid Collec-tion (accession number: LMBP 13040) and is thus publicly available.
