# Supplementary material for: Discovery of a Kojibiose Hydrolase by Analysis of Specificity-Determining Correlated Positions in Glycoside Hydrolase Family 65
Source: Molecules. 2021 Oct 19;26(20):6321. doi: 10.3390/molecules26206321 (PMC8537180; doi:10.3390/molecules26206321)
Supplement: Supplementary file 1 [file molecules-26-06321-s001.zip › molecules-1400566-supplementary.pdf]

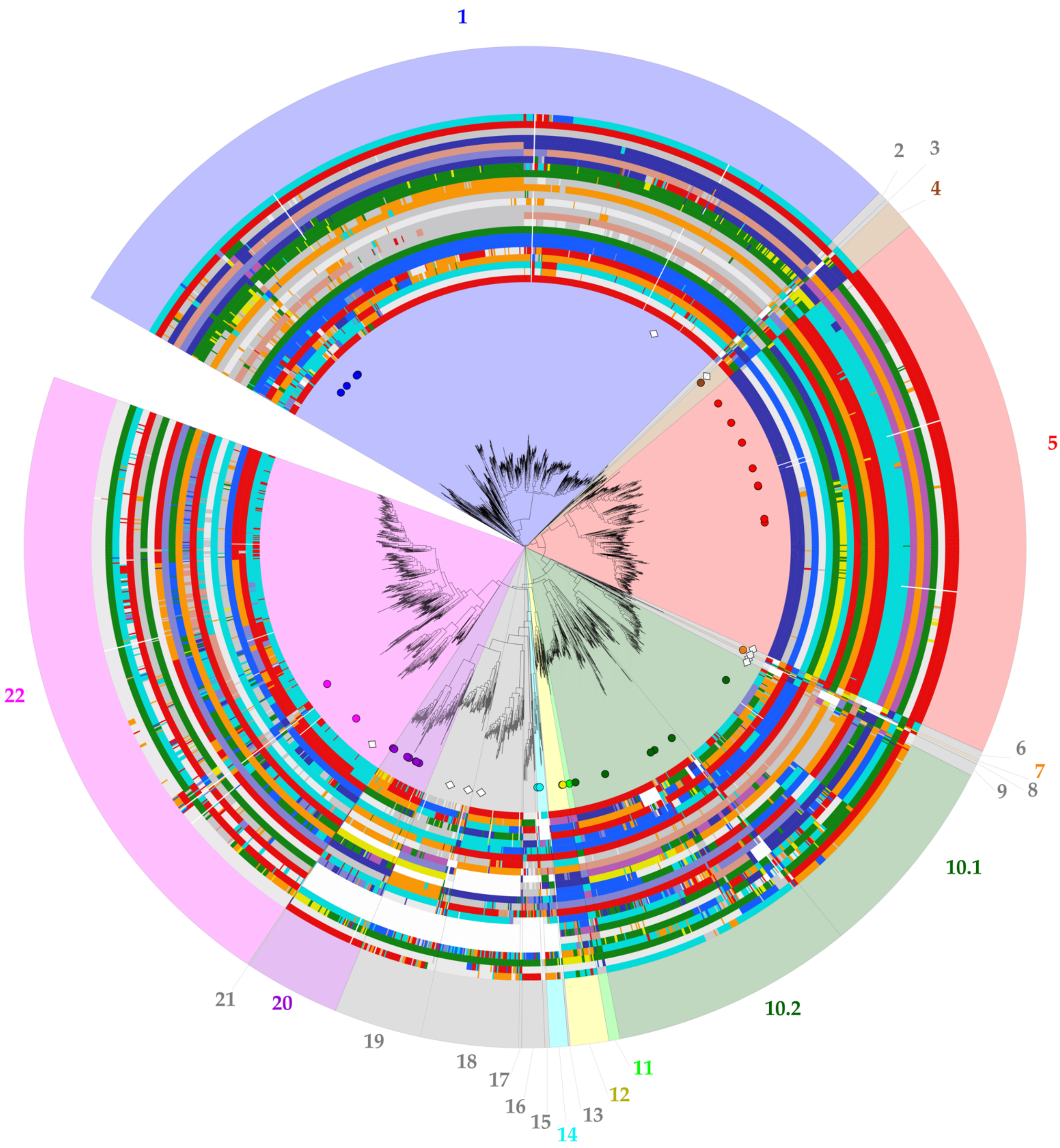

Figure S1. Phylogenetic tree of family GH65 with a visualization of the amino acids present at 24 correlated positions, shown as colored rings around the phylogenetic tree. From inside to outside, these rings represent positions 56, 62, 63, 64, 338, 378, 392, 394, 402, 416, 417, 418, 420, 485, 486, 487, 581, 584, 585, 586, 590, 603, 630 and 669 (*Cs*KP numbering). All annotated representatives and all new enzymes discussed in this study are indicated with circles and diamonds, respectively. The tree is divided into 22 subgroups, which are colored according to their putative specificity. The reader is referred to the legend of Figure 3 for the explanation of the colors and symbols used in this figure.

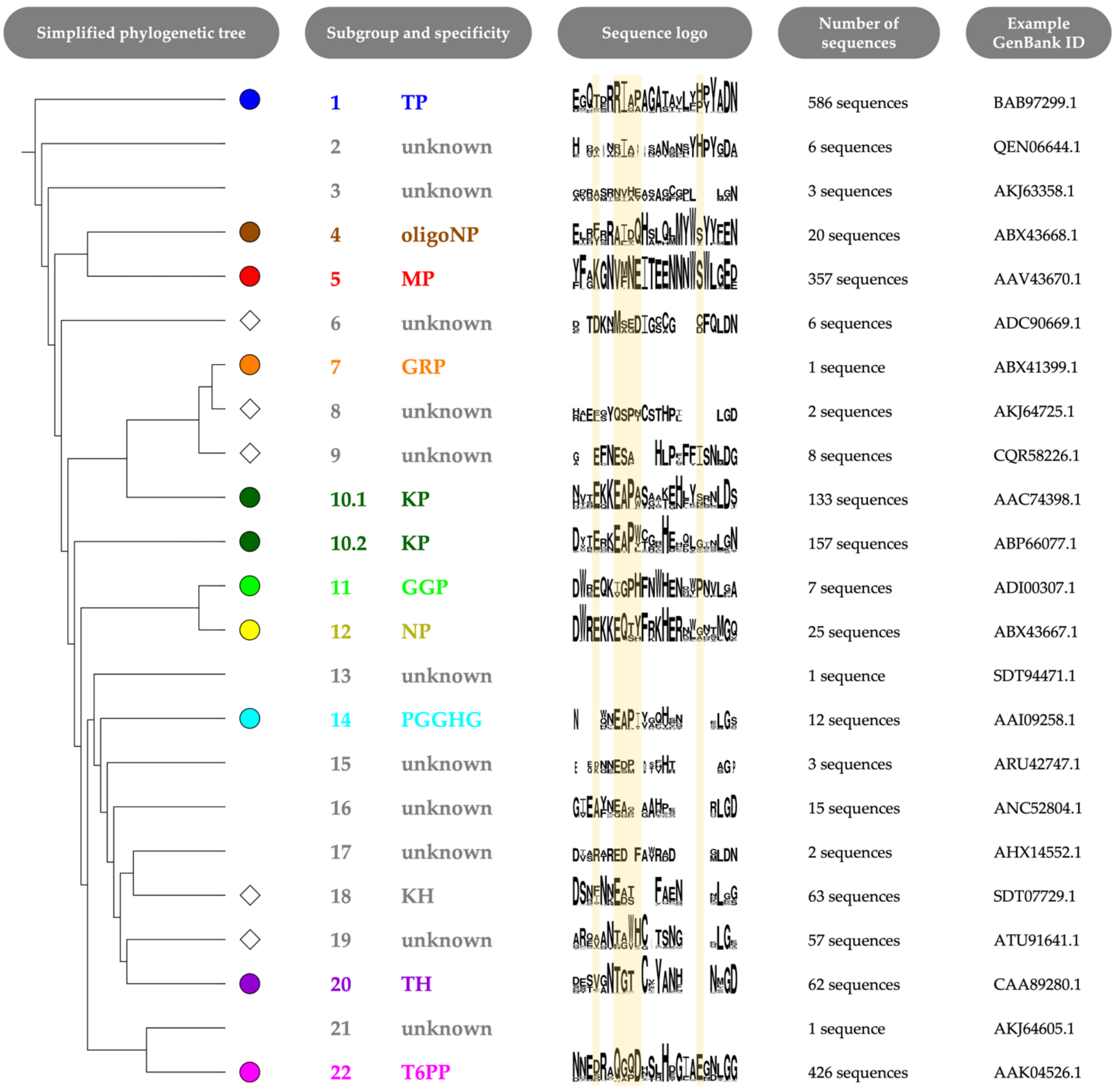

Figure S2. Simplified phylogenetic tree with sequence logo of 24 correlated positions (left to right, positions 56, 62, 63, 64, 338, 378, 392, 394, 402, 416, 417, 418, 420, 485, 486, 487, 581, 584, 585, 586, 590, 603, 630 and 669; *Cs*KP numbering). The six selected correlated positions are highlighted in yellow. A sequence logo for subgroups 7, 13 and 21 is not shown, as these branches contain only one sequence (with motifs DQISNNVSWRHPLHPY--QAHLSC, D-DAGNEAP---HAE------L-- and D--EGRQDRRDIDHTGQA---PFE, respectively). The GenBank ID of one member of each subgroup is provided as example.

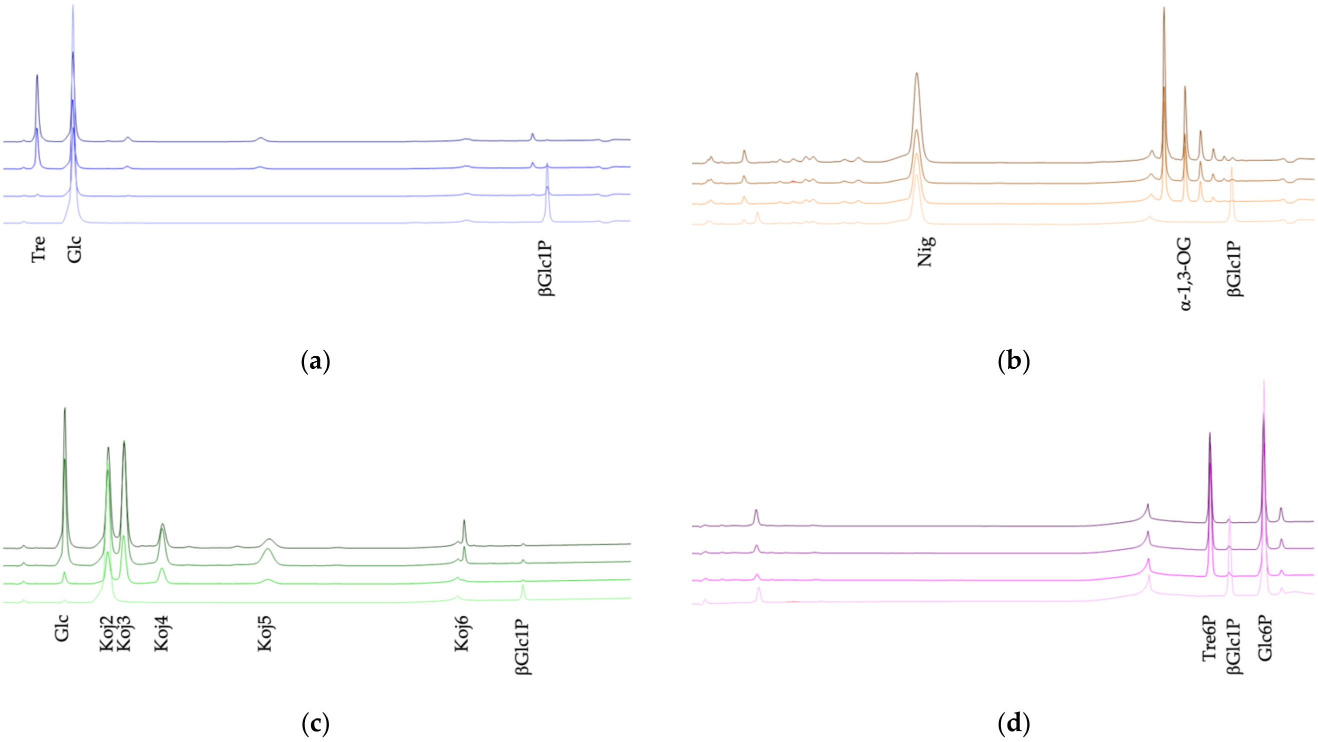

Figure S3. Reaction profile of selected GH65 glycoside phosphorylases in the synthetic direction of the reversible reaction: (**a**) Activity of the phosphorylase from *Thermobispora bispora* (*Tb*GP) on glucose; (**b**) Activity of the phosphorylase from *Caldicellulosiruptor hydrothermalis* (*Ch*GP) on nigerose; (**c**) Activity of the phosphorylase of *Halothermothrix orenii* (*Ho*GP) on kojibiose; (**d**) Activity of the phosphorylase of *Caldithrix abyssi* (*Ca*GP) on glucose 6-phosphate. Samples were taken at the start of the reaction and after 1, 6 and 24 hours (bottom to top chromatogram in overlay) and were analyzed with HPAEC-PAD. All reactions contained 10 mM βGlc1P, 10 mM of the acceptor and 0.1 mg/mL purified enzyme in 50 mM MOPS buffer (pH 7.0) at 30 °C. Glc: D-glucose, Glc6P: D-glucose 6-phosphate, Koj2: kojibiose, Koj3: kojitriose, Koj4: kojitetraose, Koj5: kojipentaose, Koj6: kojihexaose, Nig: nigerose, Tre: trehalose, Tre6P: trehalose 6-phosphate, α-1,3-OG: α-1,3-oligoglucans, βGlc1P: β- D-glucose 1-phosphate.

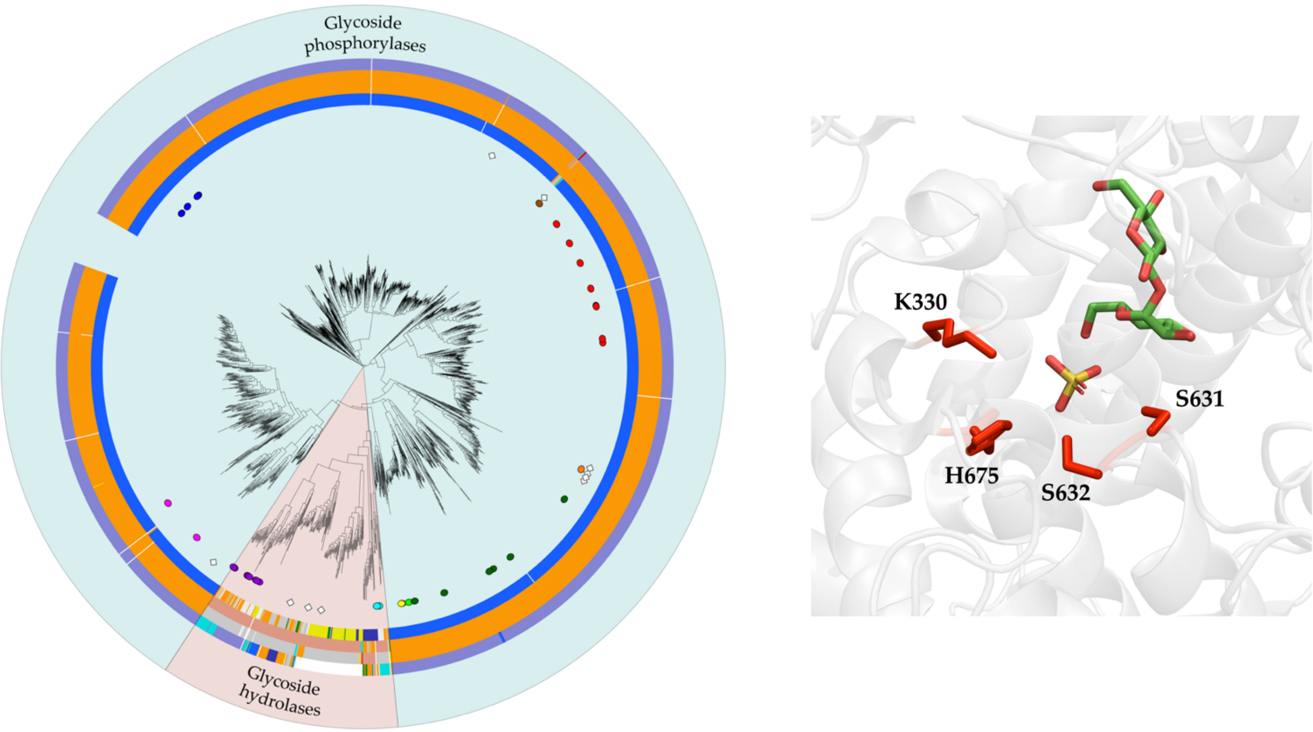

(**a**) (**b**)

Figure S4. Glycoside phosphorylases and hydrolases in family GH65: (**a**) Phylogenetic tree of family GH65 with a visualization of the amino acids present at four positions known to be involved in phosphate binding, shown as colored rings around the phylogenetic tree. From inside to outside, these rings represent positions 330, 631, 632 and 675 (*Cs*KP numbering). The reader is referred to the legend of Figure 3 for the explanation of the colors and symbols used in this figure; (**b**) Location of positions 330, 631, 632 and 675 (red sticks) in the structure of *Cs*KP (PDB ID: 3WIQ). Kojibiose (green sticks) and sulphate (yellow sticks) are also shown.

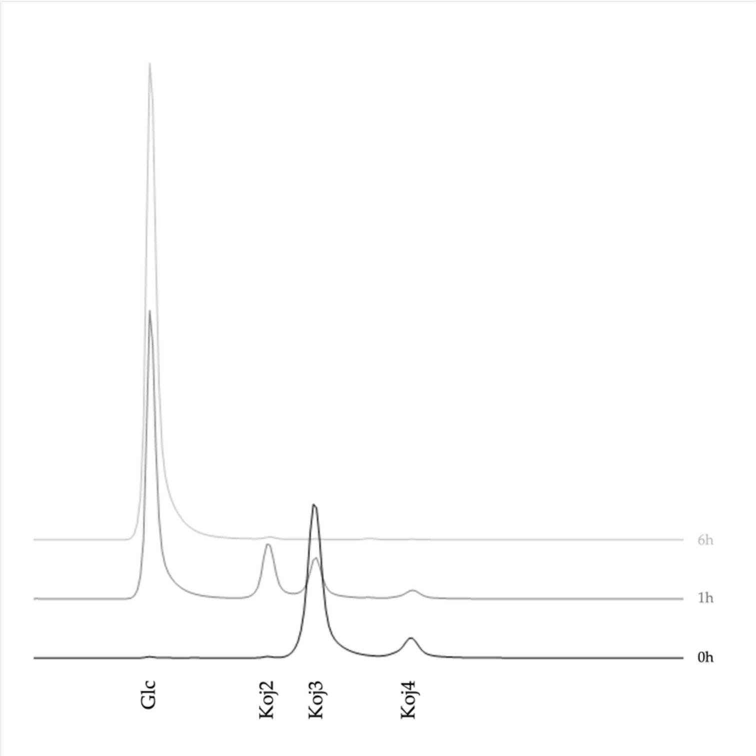

Figure S5. Hydrolysis of α-1,2-oligoglucans by *Mm*GH. The reaction was performed with ~50 mM kojitriose and ~5 mM kojitetraose and 0.1 mg/mL purified enzyme in 100 mM sodium acetate buffer (pH 4.5) at 30 °C. Samples were taken at the start of the reaction and after 1 and 6 hours (bottom to top chromatogram in overlay) and were analyzed with HPAEC-PAD. Glc: d-glucose, Koj2: kojibiose, Koj3: kojitriose, Koj4: kojitetraose.

Table S1. Acceptor profile of six GH65 glycoside phosphorylases in the synthetic direction of the reversible reaction. +++: very strong activity; ++: strong activity; +: moderate activity; +/-: low activity; -: no activity. None of the tested enzymes showed activity on fructose, lyxose, mannose, tagatose, L-rhamnose, cellobiose, lactose, lactulose, melibiose, arabitol, erythritol, galactitol, glycerol, mannitol, ribitol, sorbitol, xylitol, galactosamine, *N*-acetylgalactosamine, *N*-acetylglucosamine or *N*-acetylmannosamine.

Acceptors	*Tb*GP	*Ch*GP	*Ho*GP	*Ca*GP	*Mi*GP	*Pr*GP
Arabinose	-	-	-	-	-	+/-
Galactose	-	-	-	-	+/-	+++
Glucose	+++	-	+++	-	+/-	+++
Psicose	-	-	-	-	-	+/-
Ribose	-	-	-	-	-	++
Xylose	++	-	+	-	-	+++
L-arabinose	-	-	-	-	-	+++
L-fucose	-	-	+/-	-	-	-
L-ribose	-	-	-	-	-	+
L-sorbose	-	-	+/-	-	-	-
L-xylose	-	-	+	-	-	+
Gentiobiose	-	-	+++	-	-	+
Isomaltose	-	+	+++	-	+++	-
Isomaltulose	-	+/-	+++	-	+++	-
Kojibiose	-	+++	+++	-	++	-
Maltose	-	+++	+++	-	++	-
Maltulose	-	+/-	-	-	++	-
Nigerose	-	+++	+++	-	++	-
Sucrose	-	-	+	-	+	++
Trehalose	-	-	+	-	+	-
Turanose	-	+++	-	-	+	-
α-methylglucoside	-	+/-	+/-	-	++	-
β-methylglucoside	-	+/-	+/-	-	-	-
Glucose 6-phosphate	-	-	-	+++	-	-
water	-	-	+	-	-	-

Table S2. Comparison of the substrate profile of all α-glucosidases with hydrolytic activity on kojibiose reported in the BRENDA database [64] and *Mm*GH. ++: preferred substrate for hydrolysis; +: hydrolysed substrate; -: substrate is not hydrolysed. Glc-Gal-Hyl: α-glucosyl-1,2-β-galactosyl-L-hydroxylysine, MUαGlc: 4-methylumbelliferyl α-glucoside, *p*NP: *p*-nitrophenyl.

EC	Name	Organism of origin	Kojibiose	Maltose	Malto-oligosaccharides	Isomaltose	Isomalto-oligosaccharides	Nigerose	Nigerotriose	4-α-nigerosyl glucose	Trehalose	Sucrose	Leucrose	Isomaltulose	Turanose	Panose	Isopanose	Melezitose	Amylopectin	Amylose	Dextran	Glycogen	Pullulan	Starch	β-limit dextrin	Ethyl α-glucoside	Methyl α-glucoside	*p*NP α-glucoside	*p*NP β-glucoside	Phenyl α-glucoside	α-glucosyl fluoride	MUαGlc	Methyl α-maltoside	*p*NP α-maltoside	Phenyl α-maltoside	Methyl α-maltotrioside	Maltitol	Glc-Gal-Hyl	Ref.
3.2.1.3	Glucan 1,4-α-glucosidase (glucoamylase)	*Aspergillus awamori*	+	++	++	+	+	+			-					+			+			+	+	+				+			+								[65]
3.2.1.10	Oligo-1,6-glucosidase (isomaltase)	*Parageobacillus thermoglucosidasius*	+	-		++	++	+								+												+											[66]
3.2.1.20	α-glucosidase (maltase)	*Allium fistulosum*	+	++	+	+					-	-				+			+			+	-	+			-			+					+				[67]
		Apis cerana japonica	+	+	+	-		-				++												-				+											[68]
		Apis mellifera	+	++	++	-		+			-	+			+	-								-			-	+		+					+				[69]
		Aspergillus nidulans	+	++	++	+	+	+			+	+				+								+				+											[70]
		Aspergillus niger	+	++	++	+		+											+	+	-	-	+	+						+					+				[71]
		Bifidobacterium longum	+	+	++	+		+			-								-	-				-				+											[72]
		Entamoeba histolytica	+	+		+		++			+																					+							[73]
		Equus caballus	+	++	++	+		+			-	-												+				+											[74]
		Mortierella alliacea	+	+	+	+		+			+	+							+	+	+	+	+	++		+		+						+			+		[75]
		Mucor javanicus	+	++	+	-		+			-	-			-	-				+	-			+			-			-			+		+	+	-		[76]
		Oryza sativa	+	++	++	+		+								+			+		-	+		+	+					+					+				[77]
		Panicum miliaceum	+	+	+	+		+											++	+				+						+									[78]
		Purpureocillium lilacinum	+	+	+	+		++		+	-	-								+	-	+	-	+				+											[79]
		Schizosaccharomyces pombe	+	++	++	+		+								+								+						+					+				[80]
		Sulfolobus acidocaldarius	+	++	++	+	+	+			-					+						-		+				+											[81]
		Sus scrofa	+	++	++	+		+			-	-		+	+	+	+		+	+		+		+			+	+	+										[82]
		Thermoplasma acidophilum	+	++	++	+	+	+			-					+												+											[83]
		Thermus thermophilus	+	+	+	+	+	+			++	+	+	+	+	+		-	-			-	-	-															[84]
		Zea mays	+	++	++	+		+							+	+				+				+						+					+				[85]
3.1.2.70	Glucan 1,6-α glucosidase	*Streptococcus mutans*	+	+	+	++	++	+			-	+				++					+						+	+											[86]
3.2.1.106	Mannosyl-oligosaccharide glucosidase	*Escherichia coli*	+	+	+			++	+	+	+										-	-	-	+															[87]
3.2.1.107	Protein-α-glucosyl-1,2-β-galactosyl-L-hydroxylysine α-glucosidase	*Rattus norvegicus*	+	+		+		+			-	-			-												-	-	-									++	[45]
3.2.1.207	Mannosyl-oligosaccharide α-1,3-glucosidase	*Sporothrix schenckii*	+	+		+		++			+																					+							[88]
3.2.1.x	Kojibiose hydrolase	*Mucilaginibacter mallensis*	++	-		-		+			-	-		-	-			-																					This study

Table S3. Nucleotide sequences encoding the enzymes expressed in this work. All sequences were codon-optimized for expression in *E. coli*.

Enzyme	Codon-optimized sequence
*Tb*GP	ATGATTCGTCATCCGGCATTTGCATGTGATCCGTGGCAGGTTTGTGAAAAAACCCTGAGCCTGGATGTTCTGGCACAGACCGAAAGCATTTTTGCACTGAGCAATGGTCATA<br>TTGGTCTGCGTGGTAATCTGGATGAAGGTGAACCGCATGCACTGCCTGGCACCTATCTGAATAGCGTTTATGAACTGCGTCCGCTGCCGTATGCCGAAGCAGGTTATGGTTA<br>TCCGGAAAGCGGTCAGACCGTTGTTAATGTTACCAATGGTAAACTGATTCGCCTGCTGGTTGATGATGAACCGTTTGATGTTCGTTATGGCACCCTGCATGAACATGAACGT<br>GTTCTGGATATGCGTGCAGGTACACTGACCCGTCGTGTTCGTTGGTGTAGTCCGGCAGGCGGTCATGTTCGTATTACCAGCACACGTCTGGTTAGCTTTACCCATCGTGCAG<br>TTGCAGCAATCAAATATGAAGTGGAACCGGTTGATCGTCCTCTGCGTGTTGTTGTTCAGAGCGAACTGGTTGCAAATGAAACCGTTCCGCGTATTGATGCAGATCCGCGTGC<br>CGGTGCACTGATTCCGACACCGCTGGTTCTGGAAGAAAATATTCCGGCAGCCGAAGGTGCAGCCGTTATGGTTCATCGTACCCGTGTTAGCGGTCTGCGCGTTGCAGCAGCA<br>ATGCGTCATGAAGTTGATGGTCCGCAGGGCACCCGTATTGAAGCCGATGGTGAAGGTGATGTTAGCCGTGTTACCGTTGCAACCCGTCTGCTGGCAGGTCAGCGTCTGCGTC<br>TGATTAAATACATTGGCTATGGTTGGAGCGCAGTTCGTAGCCGTCCGGCACTGCATGATCAGGTTGTTGCAGCACTGGCAGCAGCCAAACTGACCGGTTGGGATGGTCTGTG<br>TGCAGAACAGCGTGAATTTCTGGATGAGTTTTGGGCAGGCGCAGATGTTGAACTGGAAGATGATGAAGAAGTTCAGCAGGCAGTTCGTTTTGGTCTGTTTCATCTGCTGCAA<br>GCGGGTGCACGTGTTGAACAGCGCCTGATTCCTGGTAAAGGCCTGACCGGTAGCGGTTATGATGGTCATGCATTTTGGGATACCGAAGGTTTTGTTCTGCCGGTTCTGACCT<br>ATACCTATCCTCGTGCAGCAGCCGATGCACTGGCATGGCGTCTGAGCATTCTGCCGAAAGCACGTGCCCGTGCAGCACAGCTGGGTTTAGAAGGTGCCGCATTTCCGTGGCG<br>TACAATTAATGGTGAAGAATGCAGCGGTTATTGGCCAGCAGGCACCGCAGCATTTCATGTTAATGCCGATATTGCAGATGCCGTGATTCGTTATGTTGATGCAACCGAAGAT<br>GTTGGCTTTGAACGTGATGTTGGTCTGCCGCTGCTGGTAGCAACCGCACGTCTGTGGTGTAGCCTGGGTTTTTTTGGTCCGGATGGTCGTTTTCGCATTGATGGTGTTACCG<br>GTCCTGATGAATATAGCGCAATTCGTGATAATAACGTGTTCACCAATCTGATGGCACGTCGTAATCTGCTTGCAGCCGCAGCAGCAGCGGAACGTTATCCGGCAGATGCAGA<br>ACGTCTGGGTGTTGCACCGGAAGAAATTGCAGGTTGGCGTAAAGCAGCCGGTGCAATGTATATTCCGTATGATGAACGCCTGGGCGTGCATCCGCAGCATGAAGGTTTTACC<br>CAGCACGAAGTTTGGGATTTTGCAAGCACCCGTCCGGAACAGTATCCTCTGATGCTGCATTTTCCGTATTTTGAGCTGTATCGTAAACAGGTTGTTAAACAGGCAGATCTGG<br>TGCTGGCAATGCATCTGTGTAGCGATTGTTTTACACCGGAACAGAAAGCACGCAACTTTGCATACTATGAAGCACTGACCGTTCGTGATAGCAGCCTGAGCGCATGTACCCA<br>GGCCGTTCTGGCAGCGGAAGTTGGTTTTCTGGAACTGGCCTATGCATATCTGGGTGAAGCAGCACTGATGGATCTGCGTGATCTGCAGAATAATACCCGTGATGGTGTGCAC<br>ATGGCAAGCTTAGCCGGTGCGTGGCTGGCACTGGTTGCCGGTTTTGGTGGTATGCGTGCCGATAATGGTGTTATTCGTTTTGCACCGCGTCTGCCTGCACTGCTGCGTCGTC<br>TGGCATTTCGTCTGCGTTATCGTGGTCGTCTGTTAGCAGTTGAAGTTACTCCGGAACGTACCGTTTATCGTTTACTGGATGGTCCTCCGCTGACCATGGTTCATTATGACGA<br>AGAAATTACCCTGAGTGATGAAGCAACACGTCCGACTCCGGAAGTTCGTCCGCCTGATATTGAAATTCGTCAGCCTCCGGGTCGTGAACCGCTGCCTCGTGTTCCGTTTCGT<br>GGTTGTGCAGCCCTGCCTGGTCGTTCACCGGCAGCTGGCGGTCGTCCTGGTCCTGCAGCAGGTCCGGGTGATCGTAAAGGTCCGGAAGCCGGTGGTCGTGCGGGTGATCCGG<br>AAACCGCACTCGAGCACCACCACCACCACCACTGA
*Ch*GP	ATGATCAACAAAAAAGGCAGCCGCTATGTTAAAGTTGATCCGTGGTGTATTATCGAAGAGAACTTCGATAAAAGCAATATGCGTGTTCTGGAAAGCCTGTTTACCGTTAGCA<br>ATGGTTATATTGGCACCCGTGGTTATTTCGATGAATTCTATACCGGTGATACCCATATTGGTACGTATGTTGCCGGTGTGTTTGAAGAGATTTATGAGAAACCGAGCTATAA<br>AGGTGTTCCGAATCGTACCCAGTTTGTTGTGAATAATGCAAACTGGCTGTATACCCGTATTATTGCAGATGGTGAAGAACTGGATCTGAACCATAGCAACTTTAGCGAATAT<br>AAACGTGTGCTGGACCTGAAAAAAGGTATTCTGACCCGTGAATTTATCTGGCATACCGAAAAAGGTAGCAGCTTCAAACTGAAATTTGAACGCTTTATCAGCATGACCAAAA<br>GCAATGTTTGCTGCCAGAAAATTGAAATCACCAGCCTGAATAAAAGCGGCAAAGTGAAAATTATCAGCGGTGTGGATTTTAGCCATAAACACCGTATTTATGACACCAACTA<br>TTGGGAAGGTCTGTTCAAATCCAACGAGAACGATTACATTAGCATTGGCTGCAAAACCATCAAGACCAACAAAATCAGCATTGCCAACTTTAAAATCGAGGCCAATAAAGCA<br>TGTGAACAGGGTATTGTTGAAGGCGAAAAAATCATTGCCAAAGAAATGGTGTTTGACATCGAGGAAAACGAAACCATCGAAATCGAAAAAGTGGTGGTGATCAATAGTTTTG<br>ATCGCCTGAATGAAAACCTGCAGAGCAAAAATGGTAAACTGTGTCAGAGCGTTTTTGGCCAGTATAGCTATTCGAAACTGAAACAAGAACACGAACGTTTTTGGGAACGTAT<br>GTGGGAAGAAGTGGATATTGAAATTGGTCAGGATAGCGAAAACCAGCAGGGTATTCGTTTTTGCATTTTTCAGATGCTGCAGGCATATAGCGGTATGCAGCAGGTTGTTGCA<br>GGTATTGGTGCAAAAGGTCTGAGCGGTGAAGTTTATAATGGTAACAGCTTTTGGGATAGCGAGGTTTATTGTCTGCCGTTTTACCTGTTCACCAATATTGATGCAGCAAAGA<br>AACTGCTGGAATTCCGTTATTATACCCTGCCGCAGGCACAGCAGCGTGCAAAAGAATTAGATCTGAAAGGTGCCTTTTATCCGATTGCAACCATTGATGGCACCGAAAGCTG<br>TACCCTGTGGCAGCATGCAAATCTGCAACTGCAGGTTAGCACCGCAGTTGCCTATGGTCTGTATCATTATTACATCGTGACCAAGGATGAGAAGTTCCTGTTTGAAAAAGGC<br>ACGGAAATTCTGATTGAAGTTTGTCGTATGCTGGAAAGTCGTTGTCAGCTGGGTCAGAAAGATGGTAAATATGGTTTTTTTGGTGTGATGGGTCCCGATGAATTTCACATGA<br>TGGTTAACAACGATTTCTATACCAACTATATGGCCAAGAAAAGCCTGGAATTTACCATTGAAGTGCTTAAACTGCTGAAAGCCAAAGACGAGAAACTGTATAATGAGATTAC<br>CCGCAAAACCAAACTGGAACGTAATGAAGTTGAACGCTGGGCAGATATTGCCAAAAACATGAAAATCATTCAGGATCCGCAGAGCAAGGTTTTTGAACAGCATGAAGGCTAT<br>TTTAACCTGCCGCATATTGAACTGAGCACCATTCCGGAAGATCAGATTCCGATTTACAAAAACTGGGCCTATGATCGCATCTTCCGCTATGATATGATTAAACAGCCTGCAG<br>TTCTGCTGTGTATGCTGCTGTATAGCTGTGATTTTAGCTTTGAAGAAAAAAAAGCCAACTACGATTATTACGATCTGCGCTGTATTCATGAAAGCAGCCTGAGTCCGAGCAT<br>TCATAGCATTCTGGCCTGTGAACTGGGCTATTATGATAAAGCCTATGAGTATTTTCGTTACGCAACCCGTCTGGATCTGGATAACTATAATCGTAATACCGAAGAAGGCCTG<br>CATATTACCAGCCTGGCAGCAGCATGGCTGAATATTGTTTATGGTTTTGGTGGTATGCGTAGCGATACCGCACCGATTAAACTGGCTCCGATTATTCCGGATAACTGGTCCT<br>ATTATAGCTTCCGCATTAAATACAATGGTGCCGTGCTGAAAATTGTTGTTGATCCGCAGTATGTGACCATCAAAAAACTGAAAGGCGCAGATGTTGAACTGATGGTGTATGA<br>TAAAACGTATACCATCACCGAGGACGAAATCAAAATTCCGCTGCAGAAACGTCGCCTCGAGCACCACCACCACCACCACTGA

*Ho*GP	ATGGCCATTGCCTATAATCTTGGTAAAGGCGAGTATAAAAACTGGATCATTAGCGAAACCGAGTTCAACGAAAATAACCTGCCGAAATTTGAAACCATCTTTAGCCTTGGTA<br>ATGGCTATATGGGTCTGCGTGCAGCAACCGAAGAACATTATTTCAATGAAACCCGTGGTTGTTATGTGGCAGGTATGTTTGATCGTTTTAAAGGTGAAGTTGCAGAACTGCC<br>GAATATCCCGGATTATGTTGGTATGGAAATTAAACTGGATGGCGAACGCTTTAATCTGAATCAGGGTAAAATCATTAGCTACCACCGTTACCTGAATGTGAAAGATGGTGAA<br>CTGGTTCGTGAAGTTGAATGGCAGAGTCCGGCAGGCAATATTACCAAACTGGTTTTTAAACGTTTTGTGAGCCTGGCAAATCTGCATCTGGCAGGCTTTAAAATCAAAATCA<br>TTCCGGTGAACTATAGCGGCAAAGTTGCAATTAAAACCGGCTATAATGGTCAGGTTACCAATAGCGGTGTTCAGCATTTTGTTGAAGGTGATAAACGTGTTCTGCCGGATGG<br>TAAAAGCTATCTGACCGTTCGTACCCAAGAAAGCGGTATTTTTACCATTGTTGCCGGTAAATTTCGCTTTCTGATTAACGCCAGCGAAATCAATCCGCAGCAGCAGATTGTT<br>ACCGGTCGTCGTCAGCTGTTTCTGCGTAGCGAATATGAACTGAAAGAAAACGAATGCCTGGAAATGGAAAAATGCGTGATTGTTTATACCGGTCGCGATCTGGAATTCAAAG<br>ATAAAGATATTGATAGCGGTGATATCGTGGAAACCGCACTGACCACACTGGATAAAGCAGGCACCAAACGTTATGAAGAACTGTTTAGCGAACATCGTCAGAAATGGCATAA<br>ACTGTGGCATGAAATTGATATCGAAATTGGCGGTCCGGATTTTGATCAGCTGGCAGTTCGTTTTGCACAGTATCATCTGGTTCAGATGACCCCGAGCCATGATAGCCGTATT<br>AGCGTTGCAGCAAAAGGTCTGAGCGGTGAAGGTTATAAAGGTCATGTTTTTTGGGATACCGAGATCTTTATTCTGCCGTTCTTCATTTATACCTTTCCGCAGATTGCACGTA<br>AACTGCTGGAATATCGTTATCACACCCTGGATGGTGCCCGTAAAAAAGCACGTGAAAATGGATATAAAGGTGCCATGTATCCGTGGGAAAGCGCAGATACCGGTGAAGAAAC<br>CACACCGGAATTTGGTGAAGTGGATATCAAAACCGGTAAACCGATTCGTATTTGGTGCGGTGAAATTGAACAGCATATTACCGCAGATGTTGCCTATGCAATCTGGCATTAT<br>TATCAGGTGACCGGTGATAAAGAATTCATGTATAACTATGGCACCGAAATCTTCATGGAAACAGCACGTTTTTGGGCAAGCCGTCTGGAATACAATCAAGGTCTGGATCGCT<br>ATGAAATCAAAGATGTTATTGGTCCGGATGAGTATTCCGAACATGTTAATAACAATGCCTATACCAACTATATGGTCAAGTGGCATCTGGAAAAAGCCATCGATATCTATAA<br>TTGGCTGAGTGATGATAGTCGCGACATCCTGGAAAAAATCATCAATAAAATCGCCCTGAAAGAGGATGAGCTGAACGAGTGGAAAAAAAAGAAAGACAAAATCTACCTGCCG<br>TTCCAAGAGGATAGCAAAGTGATTCCGCAGTTTGATGGCTTTATGGATCAGGATGTGATTGATATTAGCAGCTATCGTGGTGATGTTGGTGCAATTATGAAAGCATATAGCT<br>GGGATGAAATTACCAGCAGCCAGGTTATTAAACAGGCCGATGTTGTTATGCTGCTGTATCTGCTGGGTGAAGATTTTAGCCATGAAGTGAAAGAGAAGAACTACCACTATTA<br>TGAACCGAAAACACTGCATGATAGCAGCCTGAGTCCGAGCATTCATGCCATTATGGGTAAAGAAATTGGTGATCTGGATGAGGCCTATCGCTATTTTAACAAAAGCACCACC<br>ATTGATCTGGGTCGTAATATGCGTAGCTGTGATGCAGGTCTGCATAGCGCAAGCTTAGGTGGTATTTGGCAGGCAGTTGTTTTAGGTTTTGGTGGTGTGAAAGTGAAGGATA<br>ACGTGCTGAATATTGATCCGATGCTGCCGGAAAAAATGGGATTATCTGAACTTCAAACTGAAATGGCAGGGTATGCCGATTCGTGTTGAAATTCGTAATGATCGTGTTAGCGT<br>GAGCTTTCTGAATGATGATAAAGCAATGCTGAAAGACGTTTCCGTTATGGTGAAAGGTCGTAATCTGCAGCTGAATAATAACAAAGCCGTTGTGAATCTGCTCGAGCACCAC<br>CACCACCACCACTGA
*Ca*GP	ATGCCGGAATTTAAGTTTGATGCCGTTGTGTTTGATCTGGATGGTGTTATTACCCAGACCGCAAGCGTTCATAGCGCAGCATGGAAACAAATGTTTGATGAATTTCTGCGCG<br>AATATGCAGAACAGAGCGGTGAACCGTTTCGTGAATTTACCCATAATAATGATTATCTGCCGTTCGTGGATGGTAAACCGCGTTTTGATGGTGTTGCAGCATTTCTGAAAAG<br>CCGCAATATTGATCTGCCTTTTGGTGATCCGCAGGATGATCCGGGTAAAAACACAATTTGTGGTCTGGGCAATCGCAAAAACGAAATCTTTAATCGCCTGATCGATGAAGGC<br>AACCTGGTTATTTTTGATAGCACCGTGGATTTTATCAAAGAACTGCTGAAAAATGGTGTGAAAGTTGGTGTGGCAAGCAGCAGCAAAAATTGCGAAAAAATTCTGAAAGCAG<br>CCGGTCTGATCGATTTTTTTGAAACCATTGTTGATGGTCTGGTTAGCGCACGTCTGGGTCTGAAAGGCAAACCGCATCCGGATATCTTCAAAAAAGCATGCGATAATATGGG<br>CGTCGATTATCATAAAGCCATCATTGTTGAAGATGCCGTTAGCGGTGTTCAGGCAGGTCAGAATGGTCAGTTTGGTCTGGTGGTTGGTGTTGCCCGTGAAAATAATGAAATG<br>GAACTGCGTCTGAATGGTGCCGATATTGTTGTTAGCGATCTGGCAGAAATTAGCCTGAATGATCTGAATAATTGGTTTACCACCGGTCTGCAGAATGATCAGTGGACCCTGA<br>CCTATTTTAACTATGATCCGGAAAAAGAAGGTACACGCGAAGCACTGCATACCGTTGGTAATGGTTATATGGGCACCCGTGGTGCACTGGAAGAAACCAAAGCAAATGGTAT<br>TAACTATCCGGGTACATATCTGGCAGGTCTGTATAATCGTCTGACCAGCATTGTTAGCGGTCGTGAAGTTCAGAATGAAGATTTTGTTAATTGCCCGAATTGGCTGCCGCTG<br>ACCTTTAAAATCGCAAATGGCCAGTGGCTGGATCTGAACAAAGTTGAAATTATCGTGTTTGAACGTCGCCTGGATTTTCGTGATGGTAGCCTGTATCGTAAAGTGATCGTGA<br>AAGATAATAAAGGTCGCGAAACCCTGATTCAGAGCGTTCGTTTTATTAGCATGGCAAAACCTCATCTGGCAGCCCTGAAATATACCATTACACCGCTGAATTATAACAGCGA<br>TATTACCATTCGTAGCGAACTGGATGGTCAGGTTATTAATGGTGGTGTTAAACGTTATAGCCAGCTGAATAGCAAACATTGGGAAACGATTGATCAAGGTGGCGAAAAAGAA<br>AAAAGCTTTCTGGTTGTTCGTACCAACCAGAGCAAAATCAAACTGGCAGTTGCAGCACGTCTGTTCATTAAAATCAATGGTCAGCCGGTTAATAGTCCGGTGGAAATCACCA<br>CCGATCCGGGTTCAGTTAAAAGCACCCTGCAGATTAAAGGCAAAAAAAACACTCCGCTGACCGTGGAAAAAATTGTTGCAATTTATACCAGCAAAGAAGAAGATCATCCGAA<br>ACTGAGCGCAGAAAATGAAATTAATCAGGTGAACAGCTTCGAAGAACTGTACAACGAAAGCAAAAAAGCCTGGTCCAAAATCTGGAAAAAGATCGATATTCAACTGGAAGGC<br>CAGCGTCTGACCCAGAAACTGGTTCGTCTGAATCTGTATCATCTGATGATTACCGCAAGTCCGCATAATGCACAGATTGATGCAGGTATTCCGGCACGTGGTCTGCATGGTG<br>AAGCATATCGTGGTCATATCTTTTGGGATGAACTGTTTATTCTGCCGATCTATTTTATGCATTTTCCGCGTACCGCACGTAGCGTTCTGATGTATCGTTATCGTCGTCTGGA<br>TCAGGCACGTGCGTATGCAGCAGAACATGGTTATCAGGGTGCAATGTTTCCGTGGCAGAGCGGTAGTGATGGTCGCGAAGAAACCCAGACACTGCATCTGAATCCGCTGAGC<br>GGTAAATGGGATGATGATTATTCACGTCTGCAGCGTCATGTTAGCCTGGCAATTGCATATAACATCTTTAACTACTACCAGTTCACGGGTGACCTGAAATTTCTGAGTGATT<br>ATGGTGCAGAAATGTTTCTGGAAATTTGTCGTTTTTGGGCAAGCAAAGCAGATTTTGAAAGCAAATCAGGTCGCTATAATATCGATGGTGTGATGGGTCCTGATGAATATCA<br>TGAAGCGTATCCGGGTGCAAATAGCGGTGGTCTGAAAAATAACAGCTATACCAACATTATGGTTCTGTGGGCAATTGATCGTGCGTTTAACATTCTGGATCTGCTGCCGACC<br>GAAGATAAAATGAAACTGTTTAAGAAGATCGACCTGAACGAGAAAGAACTGGACGAATGGCGTAACATTAAAAACCATCTGAGCATTCCGCTGAGTCGTGAAAACATCCTGG<br>AACAGTTTGAAGGTTTTTTTGATCTGAAAGAAATCGATCTTAAAGAATATGCCCGTAAATACCGCAACATTAGCCGTATGGATCGTATCCTGAAAGCCGAAGGTAAAAATCC<br>GAACGATTACAAAGTTATCAAACAGGCAGATACCCTGATGACCTTTTATAACCTGCATGAAAACGAGATTCATCGTCTGCTGATCCAGGCAGGTTTTATGCCGAGCGAACAG<br>CTGCTGAAGAAAAACTTTGATTACTATATCAAATACACCAGCCATGGTAGCACCCTGAGCAAACTGGTGCATAGCCGTATTGCATTTCAAGCCGGTTATGAAAGCCTGAGCT<br>ATAACTATTTCATGGAATCCCTGAAAAGCGATTTCCATGATGTTCAAGGTGGTACGACCAAAGAAGGCATTCATACAGGCGTTATGGCAGGTAGCGTGCTGCTGATTCTGAA<br>AAACTATGCAGGTCTGCACTTCAAAGACGACAAAATCTATCTGGAACCGAATCTGCCTGCAGGTTGGCGTGGTATTAGCTTTTGGGTTCGTTATCAGAAACAAGAGTACCAC<br>ATCAAAATCACCCACAAGAAACTGAAAATCACCAAGACCAGTCCGGAAACCAAAATTGGTGCCGTGTATATCAAAAAGAACAAGATCCATTTCGGCGATAACAAATACGTGG<br>AAATCGAACTGCTCGAGCACCACCACCACCACCACTGA

*Mi*GP	ATGCGTCAGGATACCATTCTGAAACCGTGGCAGATTGAACTGACCACCAATAGCACCAAAGATAGCTTTGAAGAAAGCATCTTTTTTACCGGCAATGGTCGTATGGGTATTC<br>GTGGTTATCTGCCGTTTTGGCAAGAAAAACGTACCTTTGAAACCGGTCTGTTTGTTGCAGGTATTTTTGGTGAAATTAAACCGGGTATCACCGATTTTGTTAATCTGCCGAC<br>ACCGGTTTGGGGTAGCATTGAATGTGATGGTCATCCGATTACACTGAGCAGTCCGGTTCATAGCACCCTGGATATGAAAACAGGTGTTCGTAGCCAGAGCTTTCTGGTTGAA<br>AGCGGTGAAACCACACTGAATATTGAACATCGTGTGTTTTGCAGCATTAGCGATCCGAGCCTGCTGGTTCAGCAGCTGAGCTTTAGCGCAAGCCGTACCAGCGATATTACCG<br>TTCATGCAGGCATTAATACCGCCTGTTGTAATTGTCCGATTCCGGATGATCAGGTGAAAGAAAATCATGAAACCATCAAACTGGCCAAGCTGACCAATAAAGATCTGCAGAA<br>AGATAGCGTTAGCTGCACCTTTAGCATTCTTGGCACAGGTCTGATTGTGAAAGAACAAATGATTTTCCATAGCAGCTTTGCCAAAAGCGAGGATTACATTAGTGATGATAGC<br>ATTGGTAAACAGTGGTGTGGTGTTCTGCAGAGCGGTCAGAAATTTATCCTGGAAGCAACCACACGTATTACCACCAGTCGTGATATTGATAGCCTGATTGGTAGCAACGAAA<br>TCAGCGATACCTATGATGAACTGTTTGCAAAAAGCAGCCTGGCATGGGCAAGCCGTTGGAGCGAAAGCGATATTGAAATTGAAGGTGCCCCTGATGATCAGAGCGCAGTTCG<br>TTATAACATTTTTCAGCTGATTACCAGCTGTAGCGCACGTGATAGCAGCGTGAGCATTGGTGCACGTGGTCTGACCCATACACGTTATAAAGGTTGTTATTTTTGGGATACC<br>GATTTCTTCATGCTGCCGTTCTTTCTGTATACCCATCCGGAAGCAGCAAAAAGCCTGCTGATGTATCGTGTTAAAACCCTGCCGCAGGCAAAAGAAAATGCCAAAAAAATGA<br>ATAACTATGGCGCACGTTATCCGTGGATGACCTCATTTGATGGTAGCGAACAGTGTGAAAGCTGGGATATTGGTGCAAGCGAACTGCATATTACCGCAGATATTCCGTTTGC<br>AATGCAGCAGTATTTTAGCGCCACCGATGATGAAGATTTTTATCTGCAGGCAATGGAAGTGCTGATTGAAACCGCACGTTTTTGGTATAGCCGTTGTATTATTCATCCTGAT<br>GGTAGTGCAGATCTGATGTTTTGTAAAGGTCCGGATGAATATTGTGGCATCACCAACAATAACCTGTTCACCAATTGTATGGTGAAGTTCAACATTGATCTGGCCATTAATG<br>CAGCCCTGAAACTGAAAAAAGCAGATCCGGGTCGCTATACCGAACTGAGCCTGAGTGATGAAGAAGTACTGACCTGGCATAGCCTGCGTGATAATCTGAAAGAATGTCGTGA<br>TAGCCAGACCGGTCGCTATCTGCCGGATGAAACCTTTGCACGTCTGGAACCGGTGGATATCAAAACCCTGAAAACCGATGATAGCGCCAGTTATCATCATGTTTGTTTTGAT<br>CGTCTGCAGCGCTATCAGGTTATTAAACAGGCAGATACCCTGCTGCTGATGAGCCGTATGCCGAAAAAATTCACCTATATGGAACGTCTGAATGCCTGGGAAGATTTTGAAC<br>CGTGTTGTCTGCATGATAGTACCCTGAGCTTTGCAAGCCATGCACTGTTTGCCGCACAGAATGGTCTGCAGAGTGCAGCAGAAAAATATCTGAAAAAGGCACTGTATCTGGA<br>TCTGCATGAAGTGATGAATAACACCGGTAAAGAGGGTCTGCATCTGGCATGTCTGGGTGAAACCTGGTCAAGCATTCTGTTTGGTTTTCTGGGTGCAAATTTTGATAGCGGT<br>ATTCCGACCTTTAGTCCGAGTCTGCCGAGCAGCTGGAAAGCACTGAAAATGAACTTTTATTGGAAAGGCAGCCAGTATCAGATTGTTGTTAGCGAAGGTCGTAGCAGCGTTA<br>GTAAAGTTCTCGAGCACCACCACCACCACCACTGA
*Kg*GP	ATGACCGATGGCGAAAAAGATATTGCAGCAAGCGCAGATGCAGCAGGTTGGACCATTGAAGAACATTCATATCATCCGGCACGTGAACCGTTAGGTGGCACCCAGTATTGTA<br>TTGGTAATGGTTATATGGGTCTGCGTGGTAGCTATGAAGAACTGGGCACCAAAGATGTTCAGGGTCTGTTTGTTGCCGGTGTTTTTCATCGTCGTCTGCGTAAACAGTTTTG<br>TCGTGCAGATACCTTTTGTCGCAAACAGTATATCTTTGATGAAGAGGCAATGACCACACCGGAAGAAATTTGTGAAATTCAGAATCTGCCGGATCCGCTGTTTGTTCAGATG<br>CAGGTTCATGATCGTCCGTTTCGTCTGTGGGATGGTGAACTGCTGGAATATCGTCGTCGCCTGGATCTGCGTACCGGTGAACTGGTTCGTACCGTTCGTCGTGATGATGGCG<br>AAGGTCGTATTACCCGTTTTGAATTTCGTCGTTTTTGTGGTATGGATCGTCGTCATCTGATTATGCAGCGTATTTGTGTTACACCGGAAAATCATGATGCAGTTCTGGCATT<br>TCGTAGCGGTATTGATGCAGGTCTGAATGATACCTGTGAAGATACCATTCACGAAGAGGCAGAAGATGGTTTTACCCTGCGTAGCACCCTGCCGAGCACCGGTGTTAGCGTT<br>TTTCAGGCAGTTCGTCATCGTATTCTGGCAGATGGTCGTCCTGCAGATCCGGCATGGTCACGTGGTGAACGTCTGCGTCGTCATACCGCAAGCACCCGTCTGGAAGTTGCAA<br>AAGGTGAAACCGTTGTTCTGGAAAAAAGCTGTGCACTGTATAGCAGCCGTGATCCTGATCTGGATGATCCTGCAGAAGCAGCAGTTGAACTGGCAGATCGTGCAGCAGCCGA<br>AGGTTATGATGCAGCATTTGGTAGCCATGCCGAAGCATGGCGTCGCCTGTGGCGTGAAACCGATATTCGTATTGAAAATGGTCCGGATGATCAGCGTATGATTCGTTTTGCA<br>ATTTTTCACCTGATTATCGCAAGTCCGCATCCGGATAGCAGCGTTAGCATTGCAGCCAAAAGCCTGAGCGGTTATGGTTATGGTGGTTGTGTTTTTTGGGATGATGATATTA<br>ATCTGGCACCGTTTTATCAGTGGGCACTGCCGGAATATGCACGTAGCCATCATCGTTATCGTACCCGTATGCTGGATGCAGCACGTGAATATGCCCAGAGTGAAGGTCGTGA<br>AGGTGCACGTTATCCGTGGCAGAGCAGCGTGAGCGGTCATGAACATGCACCGGTTAGCATTAATTGTAGTCGTACCCAGATTCATGTTGTTCCGGATGTTGCACATAGCATT<br>TTTCGTTATGCAGCAACCAGCGGTGATCGTGAATTTCTGTATCGTCATGGTCTGGAAGCCGCAGTGGAAAGCAGCCGTTATATGATGCAACGTCTGGTTCGCCATGAAGCCG<br>AAGATCGTTATGAAATTCATGGTGTTGGTGGACCGGATGAATATCATCCTATTACCAACAATAACGCCTATACCAATTGGACCACCGCAGAAGTTCTGCTGCGCACCGCAGA<br>TGCCCTGGATGCACTGCGTGCCGAAGATGCCGATCGTGCCCGTGAACTGGAAGCACGTCTGCAGCTGCGTGACGGTGAACCGGCACGTTGGCGTGAAGCAGGTCAGAAAATG<br>TTTCGTCCGGTTGATGAAACCACCGGTCTGATTCCGCAGTGTGATGGTTTTTTTGATCTGCAAGATGATTGGGAACGTGTTGGTAGCGATTGGGGTGGTCCGGGTGCAGAAT<br>ATCACCGTTGTAAAGGTATTAAACAGCCGGATGTTATTCTGCTGCTGACCCTGTTTCCGGAACAGTTTGAACGTCGTCATTGGGTTGCAAACTGGGATTATTATGAACGCTT<br>TATTCTGCATGGTAGCAGCCTGAGTCCGAGCATTCATGCACTGGCAGCAGCAAAAATTGGTCTGGTTCAGCGTGCCCGTTATTACTTTCGTCTGGCAGGTAGCTTTGATTTC<br>GAGGATTATAACAATGATAGCGAAGCCGGTATTCATATTGGCAATTGTGGTGGTCTGTGGCAGGCAGCAATTTATGGTTTTGCCGGTCTGGAACTGCGTGGTGATACCCTGT<br>ATTTTGATCCGCATCTGCCTGAAGAATGGGGTGCAATTAGTTTTCGTCTGCATTTTCGTGGTCAGGCCGTTGATGCAGCCGTGCGTAGCGGTGGTGTTGAAATTCGTGCCGA<br>TGAAAGCAATCCGGCACCGGTTCGTATTGGTGGTTTTGGTCGTACCGATGAAGTTCGTGCGGGTGAAACCAAAGAACTGACAGGTCATGCAAGCCTCGAGCACCACCACCAC<br>CACCACTGA

*Pr*GP	ATGATGAACCGTAAAATGACCCTGGGTAAAAAAACCGGTGAAACCGAAGGTCTGCAGATGAATGAAGAAAATCTGTGGTGTCTGAAAGAGGATGGTTATCATCCGGGTCTGC<br>ATAAACATTATGAAGGTCTGTTTACCCAAGGCAACGGTTATATGCATGTTCGTGGTAGCTTTGAAGAAGGTTGTAGCGACGCACCGCAGGATGAAGAGTATCTGCGTTTTCC<br>TGAAAATGTGACCCTGGAAAAACCGCGTCATCCGAAAAGCAAACAGGGCACCTTTATTCCGGGTATTGTTGGTCGTCATCCGCTGCTGAAAGAAGAAATTGTTAATCTGCCG<br>TATTATCTGGGCCTTGAATTTCTGAGTGCCGGTGAACGTCTGGATATGGATCGTTGTCGTATTAGCGGTTATCAGCGTTGGCTGGATCTGCGTGATGGTAGCCTGCATCGTA<br>GCGTTGTTTGGGAAACCGCAGAAGGTATGAAAATTCATTGTACCTTTACGCGCTATCTGAGCATGGCAGATACCCATCTGTGTATTCAGCAGGTTCAGCTGGAAGTTCTGAG<br>CGGTGAAGGTAGTCTGGAAGTTAAAGCAACCCTGCGTGCAGATGTTCGTACCAATGGTATGAATCATTATGCACGTATTGTTCCGAGCGCAGATAGCGAAGGTATTCTGAGC<br>CTGGAAACCATTACCGAAGAAGGCAATCAGATTTTTATGCTGAGCGCACTGGAAGCAAGCGAAGCAATTGTTTGGCATGCAGAAAATACCCGTATGACCGCAGCACTGCGTG<br>GTAAATGTCGTCTGCGTACCGGTGATCGCCTGGCAGTTAGCAAACTGACCACCGTTACCACCGATCAGGATATGGAAGAAGGTACAGTTCGCGATCGTGCACTGGCACATCT<br>GGGTCATGCACGTAATCTGGGTTTAGAACAGCTGTATAATCGTCATGCAGATCGTTGGAAAAGTAAATGGCAGCATGCCGATATTCGCATCAAAGGTGATGATGCAGCACAG<br>ATTAATGTTCGTGCAAGCCTGTATCATCTGATTCGTAGCAATGCAGAACAGGATGCACGTGTTGCAATTTGTGCAAAAGGTTATGCGGGTGAAGCATATTTTGGTCGCTATT<br>TTTGGGATACCGAAATTAACCTGCTGCCGTTTTTTCTGCACACCAATCCGCAGGCAGCCCGTAATCTGCTGCTGTTTCGTTATAACACCCTGGAAGGTGCACGTCGTAATGC<br>CCGTAATTATGGTTATCGTGGTGCACGTTATGCATGGGAAAGCAGCCTGAGTGGTGAAGAACAGTGTGCAAATTGGCAGTATGCAGATCATGAAATTCATATCACCGCAGAT<br>ATTGTGTATGCCCTGTATCACTATGTTAATGCAACCGGTGATGACGAATTTCTGGAACGTTATGGTATTGATATTCTGGTTGAAACCGCACGTTATTGGTGTGATCGTGTTG<br>ATTGGAATCGTGAAGGTTATGGTGAACTGCTGGGTGTTATGGGTCCTGATGAATATCTGCCGTTTACACGTAATAATGCCTTTACCAATCGCATGGTGAAATTTAGCCTGGA<br>ACAGACCGTTGCATGTCTGGATAGCCTGCAGAGCGGTAAACCGGATAGCTATGCAGCAGCAGCCGAACGCCTGGGTCTGCGTCCGGATGAACGTGAACTGTTTCGTCATACC<br>GCAGAAAAACTGCGCCTGCCGTATGATAGCCAGATGGAAATTGTTCCGCAGAGTGATGATTTTGCAGGTTATGCCGATGTTGAATTTGATAGCCTGTGGACCGATCGTAGCC<br>GTCCGTTTGGTCACTTTATTAGCCAAGAACGTAATTATCGTAGCAAAGCACTGAAACAGGCCGATGTTCTGGAACTGATGCTGTTATTTCCGCAAGAATTTCCGCGTGAACA<br>GCTGAAAGCAGCGTATGCCTATTATGAACCGATTACCACACATGATAGCAGCCTGTCAGTTGCAGTTCATGGTATTGTGGCAAGCTGGCTGAATCTTAAAGAAGAAGCCGAA<br>GCATTTCTGCAGCGTGTTATGGCAATTGATTTAGTCCGGAAAAAAAAGGTGCAGCCGAAGGCATTCATATTGCAAATTGTGGTGGTCTGTGGCAGCTGATTGTTTATGGTT<br>TTGCCGGTCTGGCAAGCGCAATGTGGTCAGATACCATTAAACTGCAGCCTCGTCTGCCGGAAGGTTGGGAAGAACTGAGCTTTCAGATTGTGTGGCGTGGTGAACATTATCG<br>CATTAGCGTTAATAGCGGTGCCTATGAAGTTCAGAAACTGAATGGTGGTGAAGCAGTTGCAGGTACAACCATTCTCGAGCACCACCACCACCACCACTGA
*Mm*GH	ATGGCACAGGATCCGTGGATTATCAAAGCCGATAAAATTGATCCGGCAAACTATTATGGTGTTACCGTTGCAAATGGCATGATTGGTATTGTTAGCAGCAGCGAACCGTTTC<br>AGGTTAAAAATGTTGTTCTGGCTGGTGCCTATGATATGTATGGTCGTGGTCGTGTTAGCAACTTTCTGAATTCATTTAACCTGCTGAACATGTATCTGCTGTTTAACGGTGA<br>TGATTGGGATGCAAGCAAAGTGAAAAACATGAAACAAGAGCTGGATATGCAGCATGCAAGCTTTACCACCACCTTTGATTATGGTGATGTTGCCAGCATCAAATACACCTAT<br>TATAGCCTGCGTCAGCTGCCGTTTTGTGTTCTGATGGATGTTAGCGTTACCGCAAAAAAAACCGTGAATATTACCGCAGCAAGCGTTATGCAGACACCGGATGCACTGCGTG<br>ATGTTCAGAATTATTACAATGAAGTTGATGGTCCGACAGGTCGTATTAGCCTGCTGACCAGCACCGCGAAAAGCCCGACCGGTAAACTGCAGCTGTGTGCAAGCAATGCATT<br>TATCTTTAATGAAGCAGATAGCCTGGCACCGCGTCTGATGCATGAAATGCTGGATAATAACATGCATAGCATGCGCTTTAGCAAAGAACTGGCAGCCGGTCAGACCTATAGC<br>TATTCAGTTGTTGGTAGCAGCATTACCAGCGCACATACCACCGATCCGCTGAATGAAGCCGAACGTCTGACCATTTTTGCACGTCTGCAGGGTCGTGATGGTCTGATTAAAG<br>CCCATACCAAAGCATGGGCAGAACTGTGGAAAAGCGATATTCAGATTGATGGTGAACCGCAGGCACAGCAGGATGTTCATAGTATGCTGTATCATCTGTATAGCTTTAGCCG<br>TGAAGGCACCGCCTATGCACCGAGTCCGATGGGTCTGAGCGGTAGCGGTTATAATGGTCATATCTTTTGGGATAGCGACCTGTGGATGTTTCCGGCACTGCTGGTTCTGCAT<br>CCGGAAATTGCAAAAAGCCTGATCGAATATCGTTATGAACGTCTGGCAGCAGCAAAACAGAATGCATTTGCACATAGCTTTAAAGGTGCAATGTATCCGTGGGAAAGCGCAG<br>ATAATGGCACCGAAGAAACACCGGTTGGTAGCCTGAGCGGTCCGTTTGAACATCATATTACAGCATGTGTTGCACTGGCAGCATGGAACTATTATTGCGTGACCCAGGATAA<br>ACAGTGGCTGCAAGAAAAAGGTTGGCCGATTATTAGCGCATGTGCAGATTTTTGGGCAAGCCGTGTTGAACGTAATGGTCCGGGTCAGTATGATATCAAAAATGTTATTGCA<br>GCCGATGAATGGGCTGAAGGCATTGATAATGATGCATTTACCAATGCAGCAGCCAAAGCAAATCTGCAGTGTGCAGCCCTGGCAGCAAAAGTTCTGAATGTTAAAGCAGATC<br>CGGATTGGCAGCTGGTTGCACAGAATATTCCGATTCTGAAATTTCCTGATGGCGTGACCAAAGAATTTGCCAGCTATAAAGGTGGCGGTATTAAACAGGCCGATGTTAATCT<br>GCTGGCATATCCTCTGAAAACCATTACCGATCCGGCACAGGTGAAAAAAGATCTGGAATTTTATGAAAGCCGCATTCCGAATGAAGGTACACCGGCAATGACCCAGGCAATT<br>TTTACCCTGCTGTATAGCCGTTTAGGTAATGGTGATAAAGCCTACCACTTTTTCAAAGATGCCTATGAACCGAATCTGAATCCGCCTTTTCGTGTTATTGCCGAAACCAAAG<br>GTGGCACCAATCCGTATTTTGCAACCGGTGCCGGTGGTATTATTCAGAGTCTGCTGATGGGTTTTGGTGGTCTGGATATTACCCCGAATGGTATTACCCAGGTGAAATCAAC<br>CCTGCCGAGCAATTGGAAAAGTATTACCATTACAGGTGTTGGTCCGGAAAAAAAGACCTATGTTGTGAAACTCGAGCACCACCACCACCACCACTGA
*Pz*GH	ATGGGTATTAGCACCAGCGTTGATAGCGATCACATTGTTCAGCTGGGTCGTAATGTTCCGCAGAGCGTTCCGGATAGTGATCGTACCGTTAATGATAGCGCAAATAACATTG<br>CAACCGCAGCAAAAACCGCAGGTTATGATAAACTGCTGAAAGAACATATTGATGCCTGGGAAGCAATTTGGGCAAGCAGCAGCATTACCGTTGAAAATGCACGTCTGCAGAG<br>CATTATTAACAGCGCACAGTATGAACTGCTGAGCAGCACCTATCCGGGTGCACGTGCCAGCATTCCGCCTGGTGGTCTGGCAGGCGTTGGTTATGGTGGTATGATTTTTTGT<br>GATGCAGAAACCTGGATGATGCCGTATCTGCTGCTGACCCATCCGCAGCTGGCAAAAAGCATTATTGATTATCGCTTTGATAATCTGCCGGAAGCGCGTGTTAATGTTACCG<br>ATACACGTTTTAATCAGGATCGTGGTATTCGTCCGCTGCGTGGTGCATATTTTCCGTGGGTTAGCGGTACAGGTGCAATGGGTAGCGAAGCCGGTGATGGTGTTCGTGGTCG<br>TCGTCAGCTGCATCTGCAGGCAGATATTGCACTGGCACAGCATCAGTATTATGCAGCAACCGGTGATCGTGCATTCCTGCAGAATCAGGCGTGGCCGATTCTGAGCGGTATT<br>GCAGATTTCTATACCACACGTGTTACCCTGACCGCAGGCGGTTATGACCTGGAACAGGTTACCGCACCGGATGAATATGTGGAAAATGTTAATACCGAAGCCTTTACCAATG<br>GCAGCGCAATTAAAGCACTGGATCTGGCAATTGAAGCAGGCAAAATGCTGAATGAAACCATTCCTCCGCTGTGGACCACCGTTCGTGATAATTTTGTTAAACCGATTGAACG<br>CGACGGCATCCATCTGGAATATAGCACCTTTAGCCGCTTTAATGAAAACAACCGTATTAAACAGGCCGATGTTGTGCTGCTGAAATATCCGATGGAATATCCTATGACAGAT<br>ACCCTGGCAGGTAATGATCTGCAGTATTACGCAAGTATTACCGATCCGGATGGTCCGAGCATGACCGATGCAGTTTATGCAGTTGTTACCGCAGAACTGGGTAGCTGTGATT<br>TTGATCGTTTTCTGGGTCGCAGCTATGATGTTGCCATGGGTCCGTATCATCAGTTTAACGAAACCCGTGTTCTGGATCGTAGCGCAGGTCAGCAGGGTCCGACCAACATTTT<br>TGTTACCGGTGCCGGTGGTTTTCTGCAAGGTGTTGGTATGGGTATGACCGGTTATCGTTTTCGTGATGATCGTATTGTGGTGAAACCGATTCTGCCGACCGCCATTGAAGGT<br>CAGCCTGCGACACGTGTTTTTTTCAAAGGTCTGAAATGGCAGGGTCGCGAATTTAATGTTGATATTGGTGCAACCGATACCTGGGTGACCCTGACCAAAGGTGATGCCGCAC<br>CGGTTCAGACCGGTGATAATCCGGATGAACTGGTTACCGTTACACAAGAAGCACCGCTGAAAATTCCGACACGTAAACCTGTTCAGTTTAATGCAGATGGCACCAAAACCAC<br>ACTGCCGTGTCCGGCACCGCAGCCGAGCAGCCTGATGCGTCTGCAGCTCGAGCACCACCACCACCACCACTGA

*Stre*GH	ATGAGTCTGACCCGTATTAGCCTGCTGGCAAGCCTGGTTGCAGCAGCACTGGTTGCACCGCTGCCTCCGCTGAGCCATGCAAGCAGTGGTGCACCGCCTGCACCTGGTTGTG<br>GTCGTAGCGGTGAAGCAGATCCGAGCTGGGCACCAGCAAGCACCACCTTTGGTGAAGCCGAAGGTTATGATCCGTATGTTGGTAATGGTTATCTGGGTCATCGTGTTCCGGC<br>AGCCGGTGCAGGTTATGCAGCAACCGCACAGAAAACCGGTTGGCCTCTGTATACACCGCGTTATGATGGTGCATTTGTTGCAGGTCTGTTTGGTCGTGAACAGGATCTGGCA<br>GGCGGTCGTGAAGTTATTGCAGCACTGCCGAGCTGGACCACACTGGAAGTTGGTGTTGGTCCGGAAACCTATGGTAGCGGTACACCGGCAGGTCGTGTTAGCCGTTATCGTC<br>AGACCGTTCATCTGCGTTGTGGTGTTGTTGTTACCAGCCTGCGTTGGACCACCGCAGATGGTCGTGCAACCGATCTGACCTATGAAGTTCTGGCAGATCGTAGTGATGTTCA<br>TGCGGGTGCAGTTCGTCTGCGTATGACACCGCATTGGAGCGGCACCGCAACCGTTACCGGTCGTCTGGATAAACGTGGTGCACGTCGTGTGACCGTTGCAGATGATGGCACC<br>TTTCGTACCACCGGTACAGGCACCGAAGGTGCAGTTGTTCAGGCAGGTAGTGGTGAAGGCACCCATACCGTTCGTGTTACCGATGGTCGCGCATATACCTTTGAAAAATATG<br>TTGGTGTGGATACCGCACTGACCAGCCGTGATCCGCTGGCGAGCGCAAGAGCAGCAGCCCGTCGTGCAGAACGTCGTGGTTGGAGCGGTGTTCTGGCCGATAGCGCAGAAGC<br>ATGGCGTACCGATTGGGCAAGCGATATTGCAGTTCCGGGTAGCGCAGATCTGACCGCATGGCTGCGTGCAGCACGTTATGGTCTGCTGGCGAATACCCGTACCGGTAGCAGC<br>GATAGCCTGGCTCCGGCAGGCCTGACCAGCGATAATTATGCAGGTATGGTTTTTTGGGATGCAGAAACCTGGATGTATCCGGGTCTGTTAGCAACCCGTCCGGAACTGGCAC<br>GTAGCGTTGTTGAATATCGTTATCGTACCCGTCATGCAGCCCGTGCAAATGCAGAACAGCTGGGTTATAAAGGCCTGTTTTTTCCGTGGACCAGCGCAAGCCGTGGTCGCCT<br>GGATACCGAATGTCAGAGCTGGGATCCGCCTCATTGTCTGACCCAGAACCATCTGCAGGGTGATGTTAGCCTGGCAGTTTGGCAGTATTATCTGGCAACCGGTGATCGTGAT<br>TGGCTGGCAGGTCGCGGTTGGCCACTGCTGAAAGGTATTGCAGATTTTTGGGAAAGCCGTGCAACAGCCGAAGCAGATGGTGGTTATAGCGTTCGTAATGTTGCCGGTCCGG<br>ATGAATATAGCAATGGTGTTGATGATGGTGTGTTTACCAATGCAGTTGCAGTTCTGGCCCTGCGTAATGCAACCCGTGCCGCACAGCTGCTGGGTGAAAGCGCACCAGCAGG<br>TTGGACCCGTGTTGCGGATGGCCTGCGTATTCCGTATGATGCCGAACGTAAACTGTTTCTGCAGTATGCAGGCTATGATGGTAGCACCATTAAACAGGCAGATACCGTTCTG<br>CTGACCTATCCGCTGGAATGGCCGATGGAAGAAGGTGCCGCAGCAGCAACCCTGGATTTTTATGCCGCACGTACCGATCCGGATGGTCCGGCAATGACCGATAGCGTTCATG<br>CAATTGATGCAGCAGCCATTGGTGAACCGGGTTGTAGCACCTATACCTATCTGCAGCGTAGCGTGCGTCCGTTTGTTCGTGGTCCGTATCACCTGTTTAGCGAAGCACGTGG<br>TGAAAAAAGCGGTGCACAGGATCCGCTGAGTGGTTTTCCGGCAGAAGATTTTCTGACCGGTAAAGGTGGTTTTCTGCAGGTTTTTACCCATGGCCTGACCGGTCTGCGTCTG<br>CGTGAAGATGGTGTGCGTCTGGATCCGAGCCTGCCTCCTCAGCTGCGCGAAGGTGTTGAACTGACAGGCCTGCGTTATCGTGGTCGTACCTATGATGTGAGCATTGGTCCGC<br>GTACCACCACCGTGCGTCTGACAGATGGCGAACCGTTTACCGTTCATACCCCTGCAGGTCCGCGTCGTCTGGCAGGTACACTGACCCTGCCGACACGTCGTCCGGATCTGAC<br>CCCGACCAGTGATGCAGCGCGTTGTCGTCCGGCAGCAGCGACAAGTGAAGCACCTGGTCTGTATGCAGCGGCAGCAGTTGATGGTAGTCCGGCAACCGCATGGTCACCGCAG<br>GGTGCGACCGGTACGCTGACCGTTGATCTGGGTCGTGCGGTTCGTGTTGCAAGCGTTACTCCGGCATGGTCAGATGTTGCACCGGCAAGCTATACCGTTGAAACCAGTCCGG<br>ATGGCCGTACCTGGCGTGCATTTCGTGCCGGTGATGTTGCCCGTAAAGTTCGTATGACCGTTACCTCAGATGATCCGGAAAAACCGGTGGGTGTGACCGAACTGGCCGTTGG<br>TGCAGAAAAACCGCTCGAGCACCACCACCACCACCACTGA

Table S4. Overview of all compounds tested as possible acceptor for the selected GH65 glycoside phosphorylases.

Monosaccharides	Disaccharides	Sugar alcohols	Other
Arabinose	Cellobiose	Arabitol	α-methylglucoside
Fructose	Gentiobiose	Erythritol	β-methylglucoside
Galactose	Isomaltose	Galactitol	Galactosamine
Glucose	Isomaltulose	Glycerol	Glucose 6-phosphate
Lyxose	Kojibiose	Mannitol	*N*-acetylgalactosamine
Mannose	Lactose	Ribitol	*N*-acetylglucosamine
Psicose	Lactulose	Sorbitol	*N*-acetylmannosamine
Ribose	Maltose	Xylitol	Water
Tagatose	Maltulose		
Xylose	Melibiose		
L-arabinose	Nigerose		
L-fucose	Sucrose		
L-rhamnose	Trehalose		
L-ribose	Turanose		
L-sorbose			
L-xylose			
